# Duodenal acidification induces gastric relaxation and alters epithelial barrier function by a mast cell independent mechanism

**DOI:** 10.1038/s41598-020-74491-1

**Published:** 2020-10-15

**Authors:** Hanne Vanheel, Maria Vicario, Dorien Beeckmans, Silvia Cocca, Lucas Wauters, Alison Accarie, Joran Toth, Hans-Reimer Rodewald, Gert De Hertogh, Gianluca Matteoli, Guy Boeckxstaens, Jan Tack, Ricard Farre, Tim Vanuytsel

**Affiliations:** 1 Translational Research Center for Gastrointestinal Disorders, Department of Chronic Diseases, Metabolism and Ageing, KU Leuven, Herestraat 49, Box 701, 3000 Leuven, Belgium; 2 Digestive Diseases Research Unit, Department of Gastroenterology, Institut de Recerca Vall d’Hebron, Hospital Universitari Vall d’Hebron, Universitat Autònoma de Barcelona, Barcelona, Spain; 3 Department of Digestive Diseases, Campus Bio-Medico University, Endoscopy Unit-Azienda Ospedaliero Universitaria di Modena, Rome, Italy; 4 Centro de Investigación Biomédica en Red de Enfermedades Hepáticas y Digestivas (CIBERehd), Instituto de Salud Carlos III, Madrid, Spain; 5 Department of Gastroenterology and Hepatology, University Hospitals Leuven, Leuven, Belgium; 6 Division of Cellular Immunology, German Cancer Research Center, Heidelberg, Germany; 7 Department of Pathology, University Hospitals Leuven, Leuven, Belgium; 8 Present Address: Department of Gastrointestinal Health, Société des produits Nestlé S.A., Nestlé Research, Vers-chez-les-Blanc, 1000 Lausanne 26, Switzerland

**Keywords:** Small intestine, Tight junctions, Translational research, Functional dyspepsia

## Abstract

Duodenal hyperpermeability and low-grade inflammation in functional dyspepsia is potentially related to duodenal acid exposure. We aimed to evaluate in healthy volunteers the involvement of mast cell activation on the duodenogastric reflex and epithelial integrity during duodenal acidification. This study consisted of 2 parts: (1) Duodenal infusion of acid or saline during thirty minutes in a randomized, double-blind cross-over manner with measurement of intragastric pressure (IGP) using high resolution manometry and collection of duodenal biopsies to measure epithelial barrier function and the expression of cell-to-cell adhesion proteins. Mast cells and eosinophils were counted and activation and degranulation status were assessed. (2) Oral treatment with placebo or mast cell stabilizer disodiumcromoglycate (DSCG) prior to duodenal perfusion with acid, followed by the procedures described above. Compared with saline, acidification resulted in lower IGP (P < 0.01), increased duodenal permeability (P < 0.01) and lower protein expression of claudin-3 (P < 0.001). Protein expression of tryptase (P < 0.001) was increased after acid perfusion. Nevertheless, an ultrastructural examination did not reveal degranulation of mast cells. DSCG did not modify the drop in IGP and barrier dysfunction induced by acid. Duodenal acidification activates an inhibitory duodenogastric motor reflex and, impairs epithelial integrity in healthy volunteers. However, these acid mediated effects occur independently from mast cell activation.

## Introduction

Functional gastrointestinal disorders (FGID) represent the most frequently diagnosed class of disorders in gastroenterology clinical practice^1^. Functional dyspepsia (FD) is one of the most common FGID occurring in up to 20% of the population and defined by the Rome IV criteria as the presence of dyspeptic symptoms in the absence of underlying organic, systemic or metabolic disease likely to explain the symptoms^2^. However, the pathophysiology of FD is incompletely elucidated, resulting in a paucity of effective treatment options.

Initial research focused on functional alterations of the stomach as a possible cause of dyspeptic symptoms, such as impaired accommodation, delayed emptying and hypersensitivity to distension^3^. More recent reports, however, point towards the duodenum as a central integrator in the pathophysiology of FD. One of the most consistent duodenal alterations is mucosal low-grade immune activation, mainly characterized by mast cell and eosinophil infiltration and activation^4,5^. The mechanism underlying low-grade inflammation in FD remains to be identified, but in a previous study, we demonstrated increased duodenal permeability, which was associated with low-grade inflammation^5^ and mast cell and eosinophil activation/degranulation^4^. We hypothesized that impaired barrier function allows increased transepithelial passage of luminal substances triggering an immune response, which in turn can activate sensory neurons causing symptoms or alter duodenogastric reflex pathways^6^. Mast cell activation has also been suggested to be involved in physiological conditions such as fat absorption^7^ and postprandial disruption of the migrating motor complex by cholecystokinin^8^.

Several potential players have been identified in the pathogenesis of the impaired barrier function in FD, including psychological stress, an altered bile acid pool and microbiota and acid exposure. Indeed, it has been shown that patients with FD display hypersensitivity to duodenal perfusion with acid and lipids^3,9^ and an increased acid exposure of the duodenum has been demonstrated in patients with FD^10,11^. Moreover, exogenous duodenal acid perfusion affects gastric sensorimotor function through duodenogastric reflex pathways in healthy volunteers, resulting in delayed gastric emptying, impaired gastric accommodation and hypersensitivity to gastric distension^12–16^. However, whether an increased duodenal acid exposure can also explain the observed increased duodenal permeability and low-grade inflammation in humans with FD, has not been studied.

Hence, the aim of the present study was to evaluate the effect of duodenal acid perfusion on barrier function and mast cell activation in healthy humans. Furthermore, we evaluated whether mast cell activation is required for activation of the duodenogastric reflex and acid-induced impairment of mucosal integrity by pretreatment with a mast cell stabilizer. Finally, we performed an ex vivo acid exposure study in wild type and mast cell deficient mice.

## Results

### Part 1: Duodenal acid perfusion decreases intragastric pressure, increases duodenal permeability and activates duodenal mast cells

#### Symptoms and duodenal pH

Duodenal saline/acid perfusion was performed in 10 healthy volunteers (3 men, 7 women; age 34.6 ± 4.2 years) to evaluate the effect of duodenal acidification on the duodenogastric reflex, mucosal barrier function and immune activation. No significant dyspeptic symptoms were induced during acid perfusion (all P > 0.1; supplementary Table S1). Acid perfusion significantly decreased the mean pH in the duodenum (7.29 ± 0.17 before vs. 3.94 ± 0.34 during perfusion; *P* = 0.002), whereas saline perfusion did not (7.34 ± 0.23 before vs. 7.13 ± 0.20 during perfusion; *P* = 0.25).

#### Intragastric pressure (IGP)

**Figure 1:**
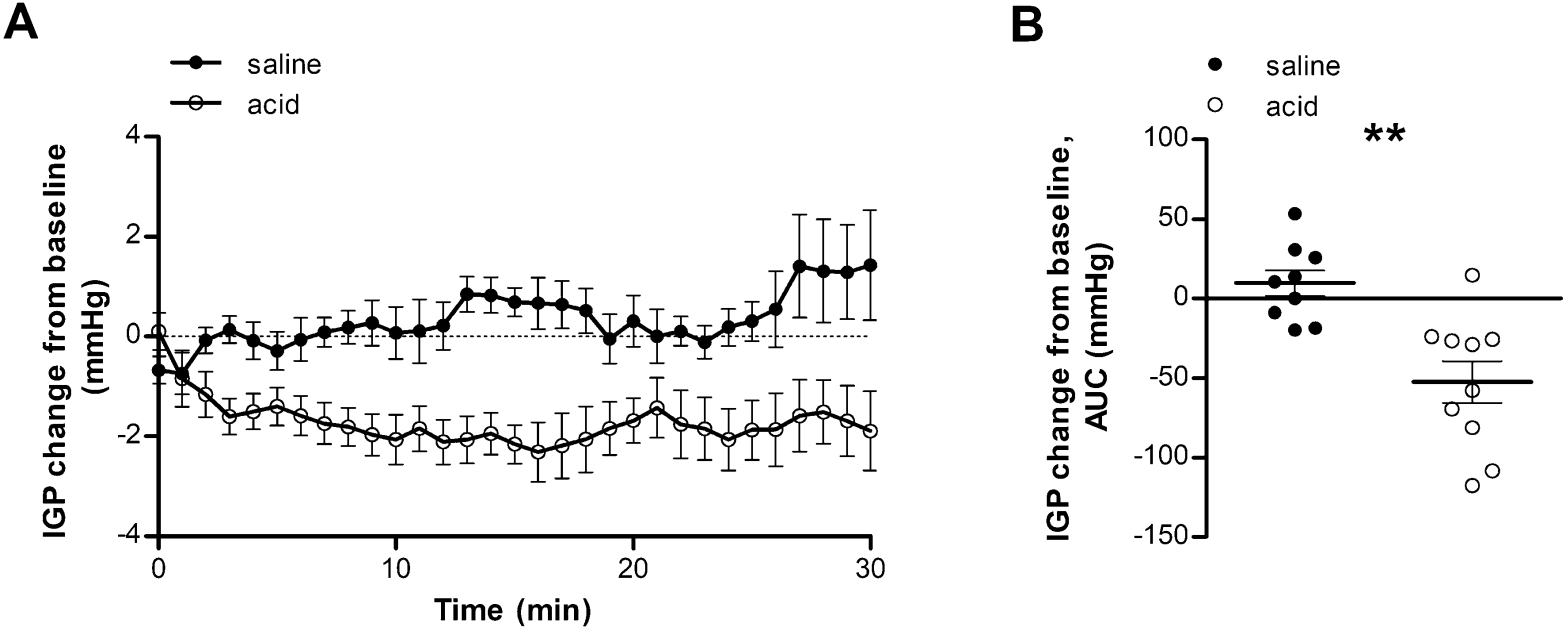
Duodenal acid perfusion relaxes the proximal stomach. IGP during saline perfusion (black dots) and acid perfusion (white dots) was evaluated using a high-resolution manometry catheter. (**A**) Time curve of IGP during saline or acid perfusion. (**B**) AUC of IGP during saline or acid perfusion. n = 9 for saline perfusion and n = 10 for acid perfusion. Data are mean ± SEM; **P < 0.01. AUC, area under the curve; IGP, intragastric pressure.

Perfusion with an acid solution resulted in activation of a duodenogastric reflex resulting in a relaxation of the proximal stomach, demonstrated by a decreased IGP compared with saline perfusion (AUC: − 52.4 ± 13.2 vs. 9.6 ± 8.1 mmHg; *P* = 0.003) (Fig. 1A,B).

#### Duodenal mucosal barrier function

**Figure 2:**
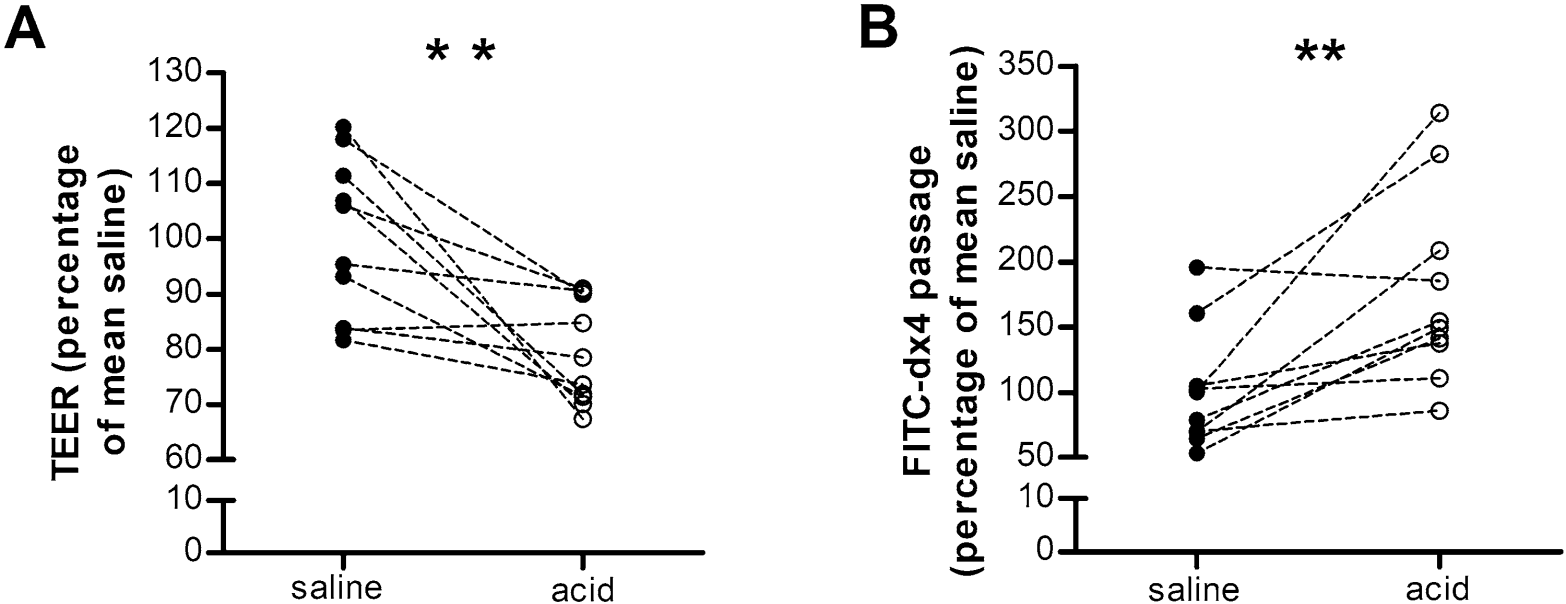
Duodenal acid impairs the mucosal barrier functions. Mucosal barrier function after saline perfusion (black dots) and acid perfusion (white dots) was evaluated in Ussing chambers by measuring TEER (**A**) and passage of FITC-dx4 (**B**). n = 9 for saline perfusion and n = 10 for acid perfusion. Results are expressed relative to the mean of the control group; **P < 0.01. FITC-dx4, fluorescently labeled dextran of 4 kDa; TEER, transepithelial electrical resistance.

To determine mucosal integrity, duodenal biopsy samples were mounted in Ussing chambers to measure transepithelial electrical resistance (TEER) and permeability to 4 kDa dextran labeled to fluorescein isothiocyanate (FITC-dx4) after duodenal saline and acid perfusion. Compared to saline perfusion, acid perfusion resulted in a lower TEER (78.9 ± 2.9 vs. 100.0 ± 4.4%, *P* = 0.005) (Fig. 2A) and higher passage of FITC-dx4 (176.9 ± 23.1% vs. 100.0 ± 14.4%, *P* = 0.007) (Fig. 2B). These data indicate that duodenal acid perfusion impairs mucosal barrier function.

**Figure 3:**
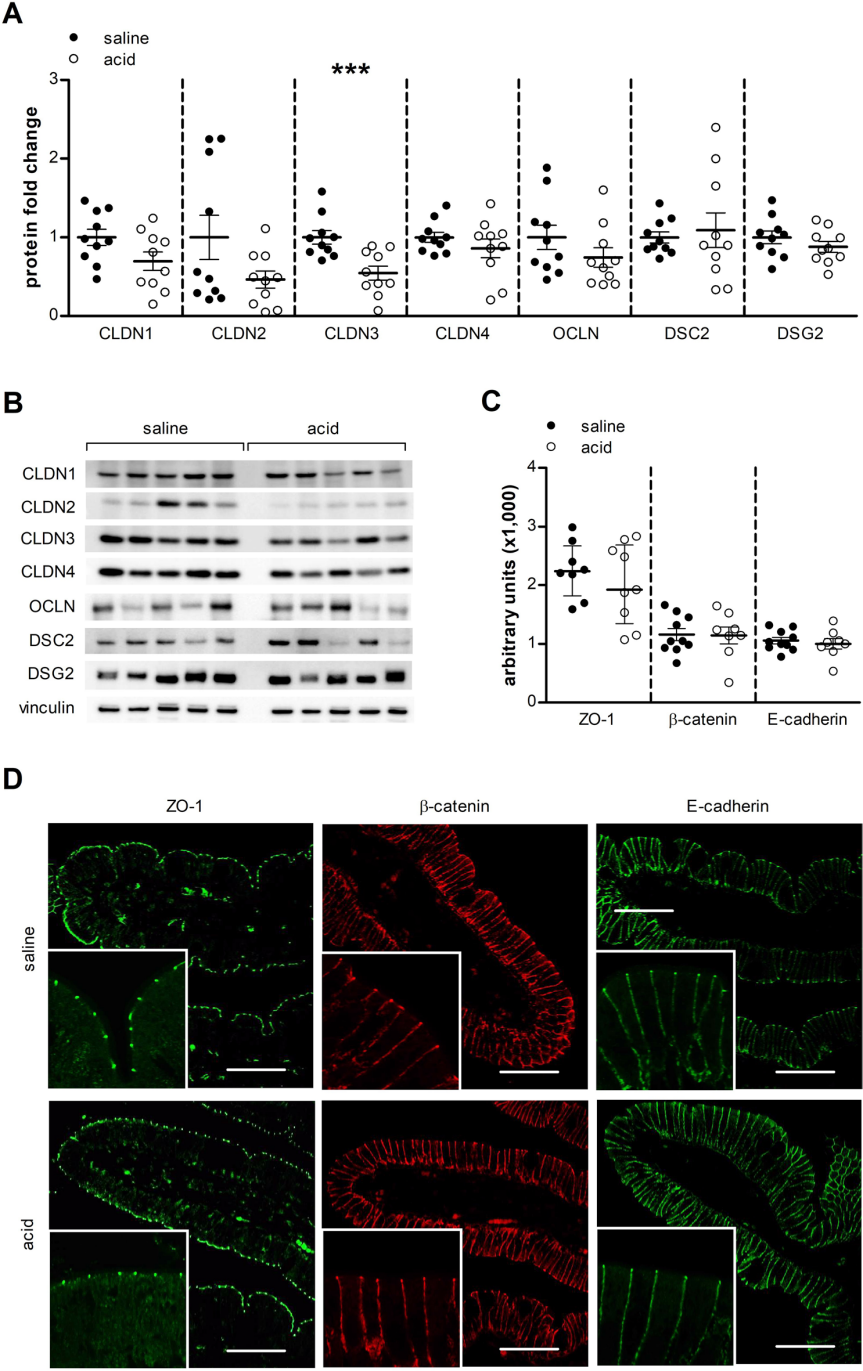
Expression of cell-to-cell adhesion proteins in the duodenal mucosa. (**A**) Protein expression of CLDN1-4, OCLN, DSC2 and DSG2 was evaluated by western blot (n = 10 for both groups). (**B**) Representative western blot of five saline perfused and five acid perfused subjects. Bands were cropped from different parts of the same gel, or from different gels. (**C**) Protein expression and localization of ZO-1 (n = 8 saline and n = 9 acid), β-Catenin (n = 10 saline and n = 8 acid) and E-cadherin (n = 10 saline and n = 8 acid) was assessed by immunofluorescence after intraduodenal saline (black dots) and acid (white dots). (**D**) Representative confocal images in mucosal biopsy specimens obtained after saline (top) and acid (bottom) perfusion. Scale bars: 50 µm. Data are mean ± SEM; ***P < 0.001. CLDN, claudin; DSC2, desmocollin-2; DSG2, desmoglein-2; OCLN, occludin; ZO-1, zonula occludens 1.

As we provided functional evidence that acid perfusion of the duodenum decreases barrier function, we investigated the expression of cell-to-cell adhesion proteins at each level of the intercellular junction after perfusion. Duodenal acid perfusion did not induce significant alterations in mRNA expression of the cell-to-cell adhesion proteins (Table 1). For the tight junction proteins, a decreased protein expression of CLDN3 (0.55-fold, *P* = 0.0006) was found after acid perfusion (Fig. 3A,B). The difference in protein expression of CLDN3 persisted after correction for multiple testing (*P* = 0.005). No difference was observed in protein expression of CLDN1 (0.70-fold, *P* = 0.12), CLDN2 (0.46-fold, *P* = 0.08), CLDN4 (0.86-fold, *P* = 0.37) and OCLN (0.74-fold, P = 0.26). Similarly, protein expression of the desmosomal proteins DSC2 (1.09-fold, *P* = 0.74) and DSG2 (0.88-fold, *P* = 0.13) was unaltered (Fig. 3A,B). Immunofluorescence analysis of the expression of the tight junction protein ZO-1 did not detect a difference between saline and acid perfusion (2261 ± 167 vs. 2027 ± 227 arbitrary fluorescence units; P = 0.56) (Fig. 3C,D). Duodenal acid perfusion did not affect the protein expression or localization at the adherens junction proteins β-catenin (1158 ± 102 vs. 1144 ± 148, *P* = 0.90) and E-cadherin (1056 ± 54 vs. 1001 ± 87, *P* = 0.90).

**Table 1:** mRNA expression of cell-to-cell adhesion proteins and eosinophil and mast cell markers.

Gene	Saline	Acid	*P* value
*CLDN1*	1.06 ± 0.14	0.93 ± 0.13	0.39
*CLDN2*	0.82 (0.65–2.08)	0.93 (0.57–1.60)	0.74
*CLDN3*	1.08 (0.87–1.19)	1.00 (0.76–1.02)	1.00
*CLDN4*	1.02 ± 0.07	1.15 ± 0.13	0.27
*OCLN*	1.03 ± 0.09	0.91 ± 0.09	0.29
*ZO1*	1.01 ± 0.05	1.07 ± 0.13	0.81
*ZO2*	1.02 ± 0.07	0.97 ± 0.10	0.84
*ZO3*	1.02 ± 0.06	0.96 ± 0.12	0.83
*β-catenin*	1.00 ± 0.03	0.93 ± 0.07	0.53
*E-cadherin*	1.02 ± 0.06	0.89 ± 0.12	0.46
*DSC2*	1.02 ± 0.06	0.95 ± 0.10	0.80
*DSG2*	1.05 ± 0.11	0.85 ± 0.15	0.18
*PRG2*	1.04 ± 0.11	0.93 ± 0.08	0.81
*TPSAB1*	2.08 ± 0.58	1.60 ± 0.56	0.20

Real-time RT-PCR was used to evaluate the gene expression of cell-to-cell adhesion proteins (upper panel) and an eosinophil marker (PRG2) and a mast cell marker (TPSAB1) (lower panel). Data are mean ± SEM or median (IQR). CLDN, claudin; OCLN, occludin; ZO, zonula occludens; DSC2, desmocollin-2; DSG2, desmoglein-2; PRG2, eosinophil major basic protein; TPSAB1, tryptase Alpha/Beta 1.

#### Low-grade inflammation

**Figure 4:**
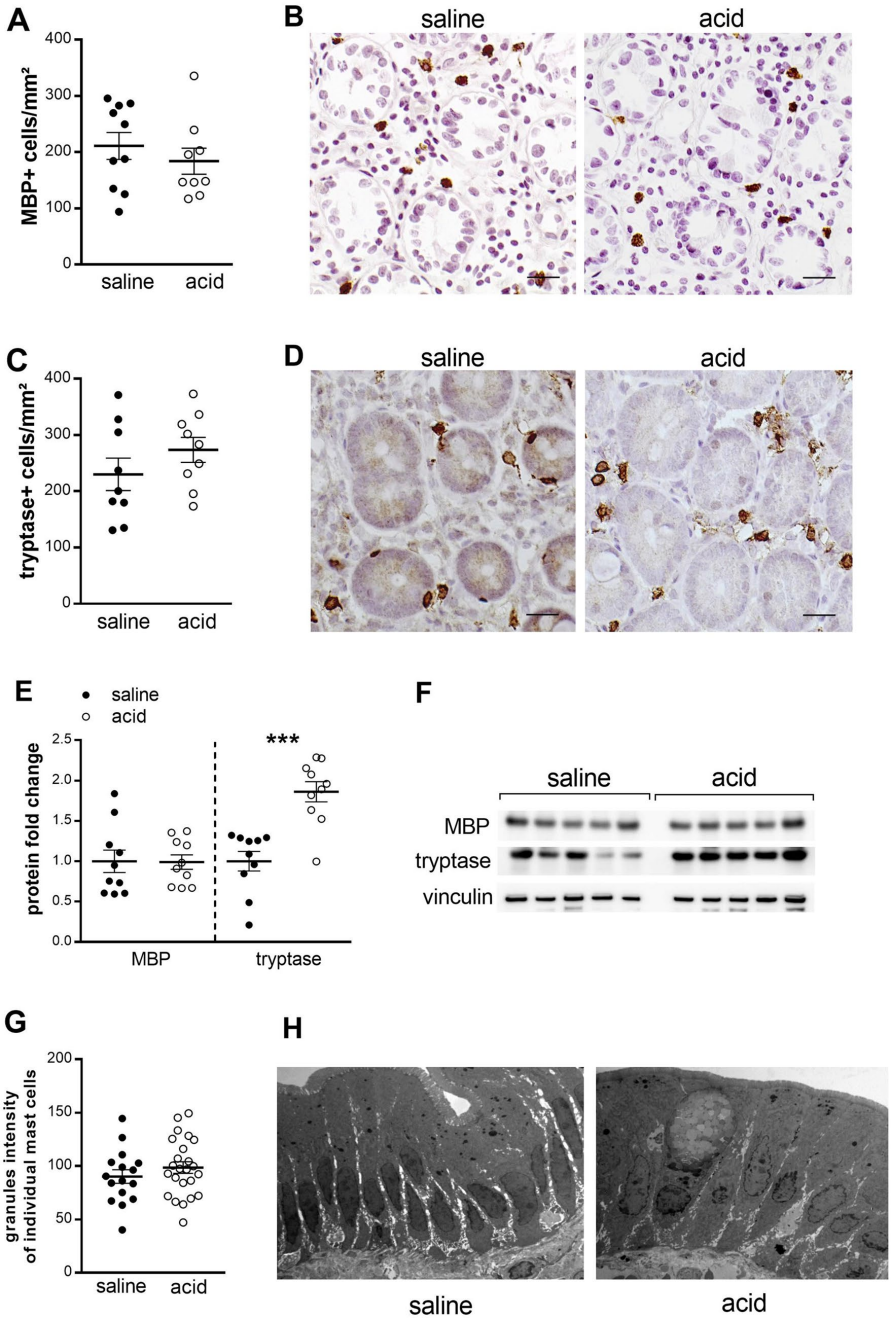
Duodenal acid induces tryptase expression, without changing mast cell counts or ultrastructure. Duodenal biopsy samples after saline perfusion (black dots) and after acid perfusion (white dots) were stained for eosinophils using eosinophilic MBP (n = 10 for saline and n = 9 for acid perfusion) (**A**) and for mast cells using tryptase (n = 9 for both groups) (**C**). Representative images of MBP (**B**) and tryptase (**D**) immunohistochemistry in mucosal biopsy specimens obtained after saline (left) and after acid perfusion (right). Scale bar: 20 µm. (**E**) Protein expression of MBP and tryptase was measured by western blot after intraduodenal saline (black dots) and acid (white dots) perfusion (n = 10 for both groups). (**F**) Representative western blot of five saline perfused and five acid perfused subjects. Bands were cropped from different parts of the same gel, or from different gels. (**G**) Electrodensity of the mast cells granules after saline and acid perfusion. (**H**) Similar ultrastructure of the duodenal epithelium after saline and acid perfusion showing no changes in cell morphology or integrity of the epithelium (×3000). Data are mean ± SEM; ***P < 0.001. MBP, eosinophilic major basic protein.

Compared to saline perfusion, no difference in eosinophil (211.0 ± 23.8 vs. 183.8 ± 23.2 MBP+ cells mm^−2^, *P* = 0.34) (Fig. 4A,B) or mast cell (230.0 ± 28.8 vs. 273.7 ± 22.1 tryptase+ cells mm^−2^, *P* = 0.34) (Fig. 4C,D) counts were detected between saline and acid perfusion. In order to exclude the possibility that degranulated mast cells may have been missed with the tryptase staining, we performed an additional quantification using an anti c-kit antibody which confirmed similar mast cell counts after saline and acid perfusion (540.0 ± 85.8 vs. 573.1 ± 60.0 c-kit+ cells mm^−2^, P = 0.75). No difference in mRNA expression of the eosinophil marker major basic protein (MBP) or the mast cell marker tryptase was found after acid perfusion (Table 1). However an increased expression of tryptase (1.86-fold, *P* = 0.0008), but not of MBP (0.99-fold, p = 0.95), was found after acid perfusion (Fig. 4E,F). These results may suggest activation of duodenal mast cells in response to acid perfusion. To explore this hypothesis more in depth, we assessed the degranulation state of mast cells (n = 16 and n = 24 respectively) by using transmission electron microscopy (TEM) in three subjects after duodenal saline and acid perfusion. The granular density of mast cells did not differ between both experimental groups (arbitrary units 90.2 ± 6.3 vs. 98.6 ± 5.4, P = 0.32, Fig. 4G). Interestingly, ultrastructural analysis of the duodenal epithelium showed that acid did not induce any damage or alteration of the epithelium, excluding a direct harmful (caustic) effect induced by acid (Fig. 4H).

### Part 2: The mast cell stabilizer DSCG does not affect the acid-induced activation of the duodenogastric reflex and the altered duodenal epithelial integrity

#### Symptoms and duodenal pH

To investigate whether mast cell activation plays a role in activation of the duodenogastric reflex and decreased mucosal integrity resulting from duodenal acid perfusion, we performed a similar acid perfusion study following a 2-week treatment with the mast cell stabilizer DSCG or placebo in a randomized cross-over fashion. For this part of the study, another group of 10 healthy volunteers (3 men, 7 women; age 23.7 ± 1.2 years) was included. There was no difference in dyspeptic symptom score during acid perfusion after treatment with placebo or DSCG (all P > 0.05; results not shown). The mean pH during acid perfusion was comparable in both conditions (7.70 ± 0.12 vs. 3.96 ± 0.41 after placebo; 7.45 ± 0.09 vs. 3.89 ± 0.43 after DSCG; between groups *P* = 0.91).

#### Intragastric pressure

**Figure 5:**
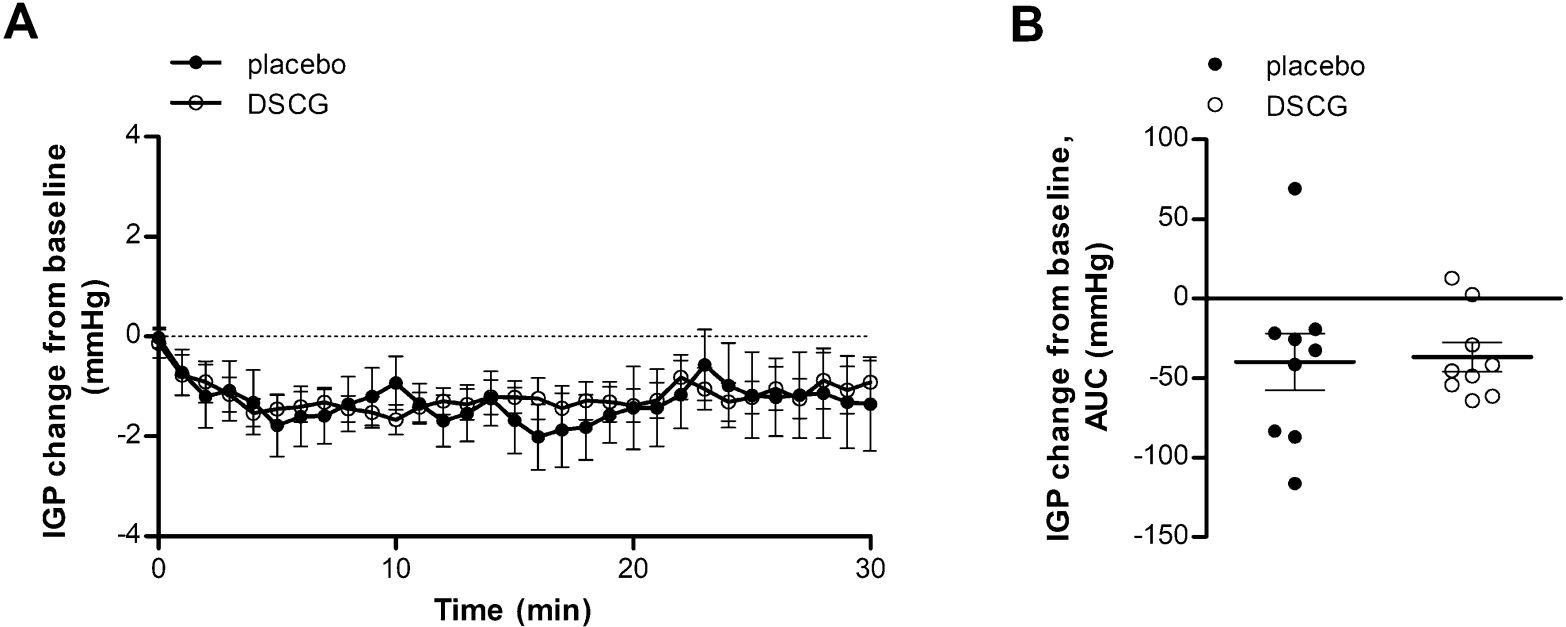
Acid-induced IGP drop is not influenced by mast cell stabilization. IGP during acid perfusion was evaluated after treatment with placebo (black dots) and DSCG (white dots) using a high-resolution manometry catheter. (**A**) Time curve of IGP during acid perfusion after treatment with placebo and DSCG. (**B**) AUC of IGP during acid perfusion after placebo and DSCG treatment. n = 9 for placebo and n = 10 for DSCG. Data are mean ± SEM. AUC, area under the curve; DSCG, disodiumcromoglycate; IGP, intragastric pressure.

Activation of the duodenogastric reflex after acid perfusion was not modified by DSCG treatment compared with placebo treatment, as both groups showed a similar drop in IGP (AUC: − 39.8 ± 17.8 vs. − 36.7 ± 9.1 mmHg, *P* = 0.86) (Fig. 5A,B).

#### Low-grade inflammation

**Figure 6:**
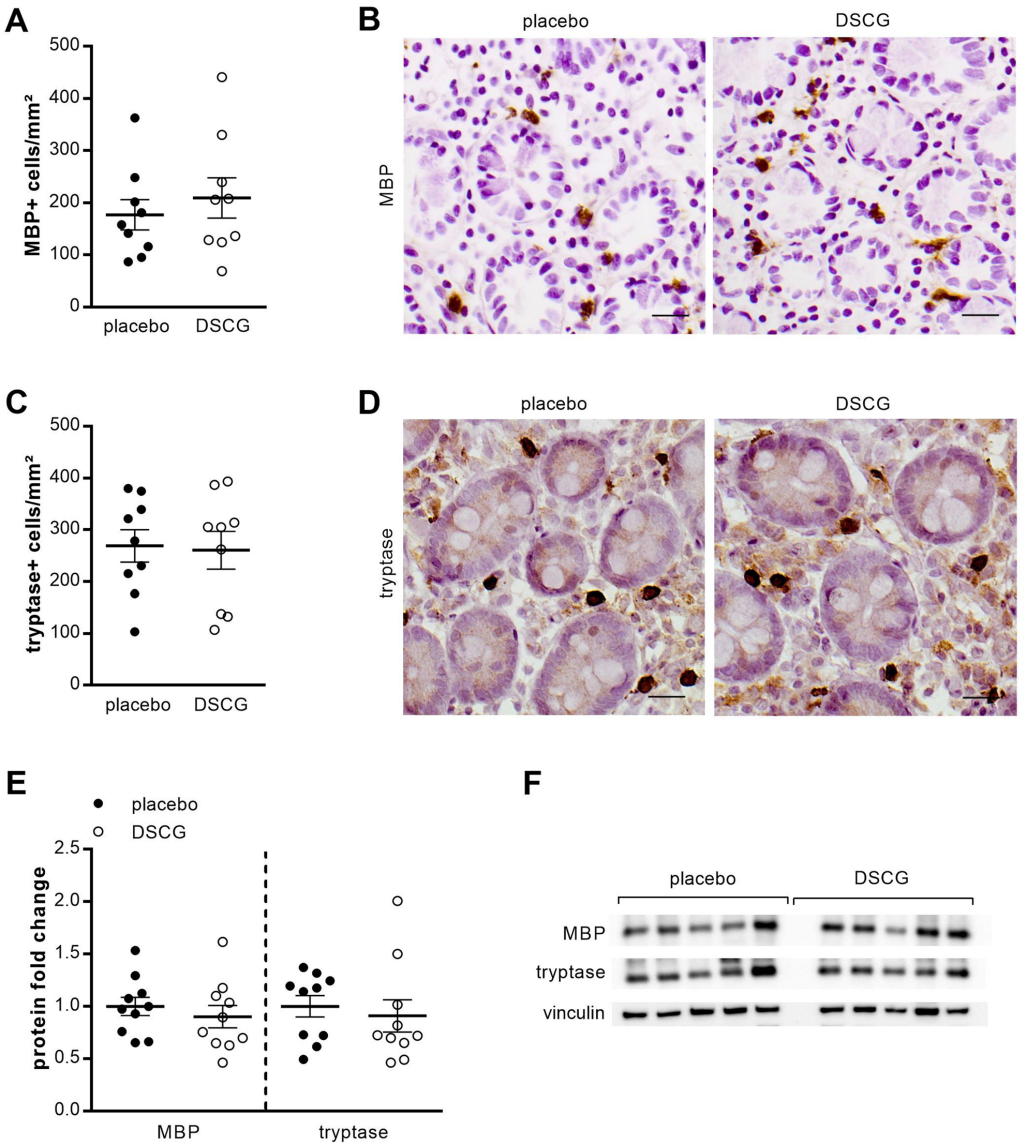
Low-grade inflammation. Duodenal biopsy samples after placebo (black dots) and DSCG treatment (white dots), were stained for eosinophils using eosinophilic MBP (**A**,**B**) and for mast cells using tryptase (**C**,**D**). Representative images of MBP (**B**) and tryptase (**D**) immunohistochemistry in mucosal biopsy specimens obtained after placebo (left) and DSCG treatment (right). Scale bar: 20 µm. (**E**) Protein expression of MBP and tryptase was measured by western blot after intraduodenal acid perfusion with placebo (black dots) and DSCG (white dots) pretreatment. (n = 10 for both groups). (**F**) Representative western blot of five saline perfused and five acid perfused subjects. Bands were cropped from different parts of the same gel, or from different gels. Data are mean ± SEM. MBP, eosinophilic major basic protein.

Eosinophil (176.9 ± 28.9 vs. 209.2 ± 38.7 MBP + cells mm^−2^, *P* = 0.53) (Fig. 6A,B) and mast cell (268.8 ± 31.5 vs. 260.5 ± 36.4 tryptase + cells mm^−2^, *P* = 0.78) (Fig. 6C,D) counts were comparable between the placebo group and the DSCG group. No difference in mRNA expression of the eosinophil marker MBP (1.26-fold ± 0.15 vs. 1.09 ± 0.14, *P* = 0.67) and the mast cell marker tryptase (1.21-fold ± 0.12 vs. 1.06 ± 0.13, *P* = 0.49) was found after DSCG treatment compared with placebo treatment. Furthermore, the protein expression of MBP (0.90-fold, *P* = 0.29) and tryptase (0.91-fold, *P* = 0.15) was similar in both groups (Fig. 6E,F).

#### Duodenal mucosal barrier function

**Figure 7:**
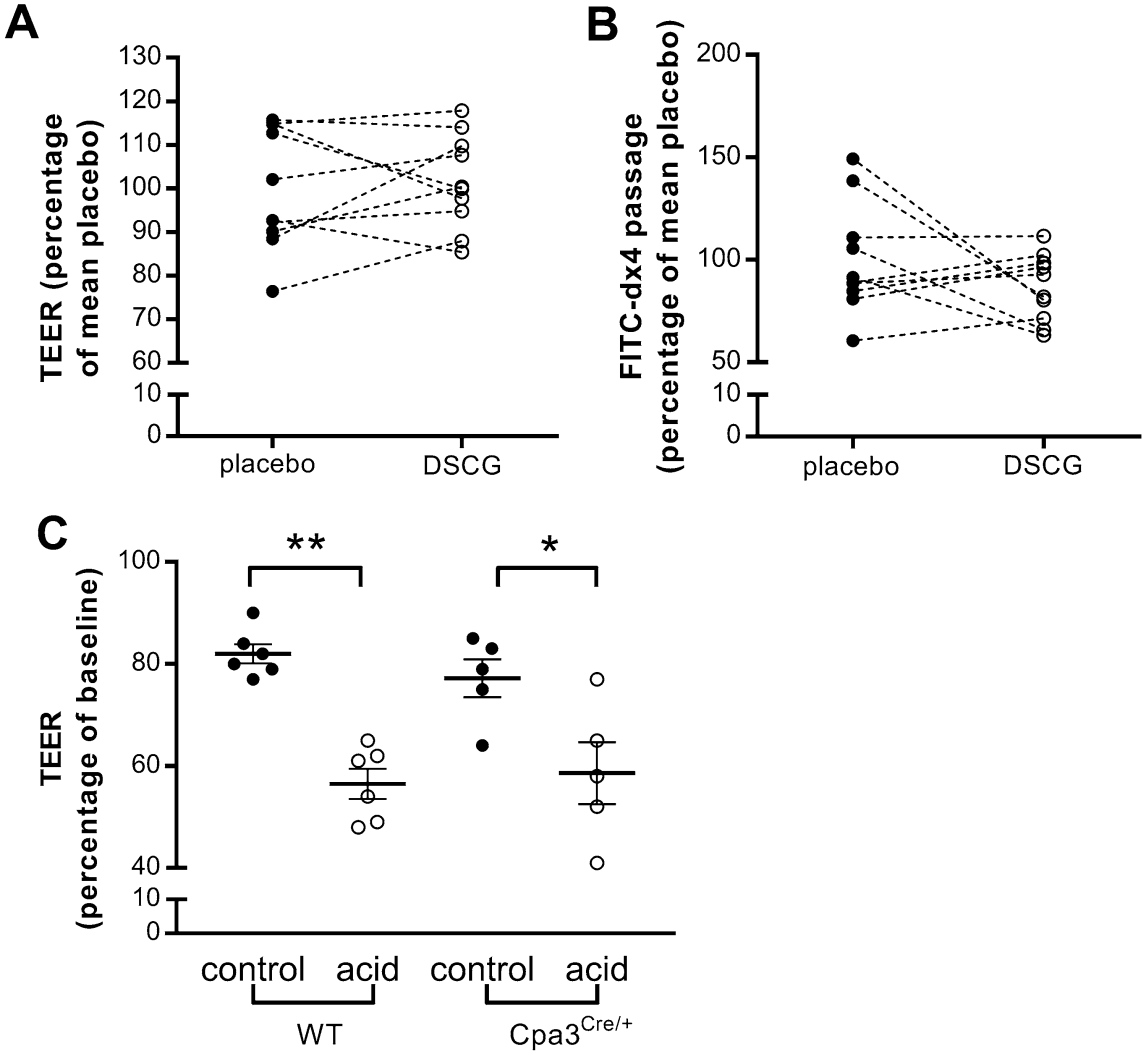
Acid-induced impairment of the duodenal mucosal barrier is not mediated by mast cells. Mucosal barrier function after acid perfusion following treatment with placebo (black dots) and DSCG (white dots) was evaluated in Ussing chambers by measuring TEER (**A**) and passage of FITC-dx4 (**B**). n = 10 for both groups. (**C**) Acid-induced reduction in TEER in wild type and in deficient mast cell Cpa3Cre/^+^ mice. Results are expressed relative to the mean of the control group. DSCG, disodiumcromoglycate; FITC-dx4, fluorescently labeled dextran of 4 kDa; TEER, transepithelial electrical resistance; WT, wild type.

There was no difference in TEER (100.0 ± 4.4% vs. 101.5 ± 3.4%, *P* = 0.70) (Fig. 7A) and passage of FITC-dx4 (100.0 ± 8.5% vs. 86.5 ± 5.2%, *P* = 0.21) (Fig. 7B) between the placebo and the DSCG group after duodenal acid perfusion, suggesting that mast cell stabilization with DSCG is not sufficient to reverse the acid-induced barrier dysfunction.

To further confirm the absence of a role of mast cells in the acid-induced alterations in the epithelial barrier function, we exposed to acid the duodenum of mice deficient in mast cells (Cpa3^Cre/+^) and their wild type littermates ex vivo. In both the wild type and the Cpa3^Cre/+^ mice, acid exposure resulted in a decreased TEER (81.0% (78.5–85.5) vs. 57.5% (48.8–62.8); 79.0% (69.5–84.0) vs. 58.0% (46.5–71.0); both p < 0.05, Fig. 7C) compared with control buffer. However, there was no difference in TEER after acid exposure between wild type mice and mast cell deficient mice. All together, these results suggest that the impaired barrier function induced by acid is not mediated by mast cells.

## Discussion

In this study, we have demonstrated that 30 min of acid perfusion of the duodenum in healthy volunteers resulted in activation of a duodenogastric reflex causing a relaxation of the proximal stomach. Duodenal acidification also impaired the mucosal integrity, shown by a decreased TEER and an increased passage of a paracellular probe that is associated with a reduced expression of the tight junction protein CLDN3. Duodenal acid perfusion additionally may lead to activation of mast cells, demonstrated by an increased expression of tryptase. Moreover, this study suggests that activation of the duodenogastric reflex and impaired mucosal integrity resulting from duodenal acidification occurs independently from mast cell activation, as pre-treatment with the mast cell stabilizer DSCG did not influence changes in IGP and barrier function in healthy controls, and a similarly impaired barrier function was found in mast cell deficient and wild type mice.

The wide variety of symptoms observed in patients with FD suggests the involvement of multiple pathophysiological mechanisms^3^. Although earlier studies reported abnormalities in gastric sensorimotor function in the pathophysiology of FD, later studies have also shown functional and structural alterations of the duodenum^3^. It has been demonstrated that FD patients display an increased duodenal acid exposure during the daytime and postprandially^10,11^, even though they are reported to have normal gastric acid secretion^17^. Excessive duodenal acid exposure might be important in the origin of dyspeptic symptoms, as a subset of patients with FD benefits from anti-secretory therapy with proton-pump inhibitors^18^. The mechanism underlying increased duodenal acid exposure in FD is suggested to be at least in part attributable to delayed duodenal acid clearance as FD patients display decreased duodenal motor activity in response to acid perfusion^19–21^.

Esophageal acid perfusion in healthy humans provoked decreased esophageal mucosal barrier function^22,23^. Other studies have demonstrated that acid perfusion of the duodenum in rats increased duodenal permeability^24,25^. We now translated these findings to humans, showing that acid perfusion of the duodenum during 30 min in healthy volunteers results in reduced TEER and increased passage to a paracellular probe, indicative of impaired duodenal barrier function. In addition, we detected a decreased expression of the tight junction protein CLDN3 in duodenal biopsy samples after acid perfusion. Claudin-3 is a sealing or barrier-forming claudin of the tight junctional protein complex. Transfecting MDCK II cell lines with human claudin-3 increased TEER and decreased permeability to a 4 kDa dextran^26^. Based on these limited data, the reduced claudin-3 expression may explain the observed barrier defect in our study, although it cannot be excluded that other, not measured, components of the junctional complex are involved as well. A decreased expression of CLDN3 had already been shown in a rat model with chronic acid-induced esophagitis^27,28^ and in an airway epithelium cell line exposed to acid^29^. These results suggest that CLDN3 expression could be a specific indicator for excessive acid exposure. Although the patients with FD of our previous study^5^ did not present with a decreased expression of CLDN3, this does not necessarily imply that excessive duodenal acid exposure is not a potential pathophysiological mechanism in this disorder. FD is a heterogeneous disorder, so it is conceivable that different pathways resulting in impaired intestinal barrier function are activated in different subgroups, underlying the varied expression profiles of the tight junction proteins. Not all FD patients present an increased spontaneous duodenal acid exposure and it is possible that other factors (e.g. stress) contribute to reduced intestinal integrity through alterations in the expression of other tight junction proteins. It is also possible that with a chronic exposure to duodenal acid—unlike the acute exposure that has been used in our study—the expression of other tight junction proteins is affected. Furthermore, a reduced expression of CLDN3 can also be a feature of decreased epithelial integrity in an earlier phase and may trigger a cascade of events that lead to other molecular abnormalities. For example, reduced CLDN3 expression can lead to increased permeability, resulting in more pronounced activation of immune cells, which in turn alters the expression of other cell-to-cell adhesion proteins including tight junctions.

Besides an altered duodenal barrier function, we also reported that duodenal acid perfusion leads to an increased expression of tryptase in the mucosa, which may be indicative of mast cell activation. This finding may confirm earlier studies in the opossum, where it has been shown that intraluminal esophageal acid perfusion is associated with mast cell activation^30–33^. We did not detect an increased number of mast cells or eosinophils after acid perfusion, unlike what we previously described in patients with FD^4,5^. This could be explained by the short time span between the acid perfusion and obtaining biopsy samples to observe an actual increased infiltration of immune cells in the lamina propria. Within this time frame, we aimed to mimic duodenal conditions of FD patients^11^ that enabled us to identify degranulation of mast cell, potentially related to an acid luminal content. Unfortunately, the experimental setting did not allow us to demonstrate significant degranulation, presumably due to the acute versus chronic exposure as happens in FD patients and the absence of additional immune and/or non-immune stimuli driving mast cell activation and degranulation^34^. Nevertheless, additional research should focus on chronic exposure to clarify the role of mast cells and other mast cell mediators besides tryptase such as histamine, within the duodenal acid environment in FD.

Because of the bidirectional communication between mast cells and neurons in the gastrointestinal tract^35,36^, which might mediate a possible effect of mast cell activation on gastric motility—we evaluated whether mast cell stabilization could prevent the decrease in IGP during duodenal acidification. Our results showed a similar drop in IGP during acid perfusion after treatment with the mast cell blocker compared with placebo, suggesting that mast cells are not involved in activation of the duodenogastric reflex during duodenal acidification. It is also currently unknown whether increased duodenal permeability occurs because of direct contact with acid or if it involves an indirect mechanism, perhaps triggered by mast cell activation. Impaired intestinal integrity and inflammation have already been shown to be closely related, but a cause-consequence relationship between these alterations has not been established and is the subject of ongoing discussion. Mast cell activation has been shown to play a key role in impaired epithelial barrier function^37–40^. Moreover, a previous study of our group observed increased small intestinal permeability after acute psychological stress in healthy volunteers, which was prevented by the mast cell stabilizer DSCG^41^. These results suggest that mast cell activation can be a pivotal element in the disruption of intestinal barrier function. Nonetheless, the opposite explanation—i.e. that impaired barrier function results in mast cell activation—remains possible as studies in animal models have demonstrated attenuation of inflammation after prevention of elevated intestinal permeability^42,43^.

Our study shows that pretreatment with the mast cell stabilizer DSCG does not block acid-induced epithelial barrier dysfunction, suggesting that impaired duodenal integrity after acid perfusion is a primary consequence of acid perfusion, and not a mast cell-dependent mechanism. Moreover, the lack of effect of DSCG on the drop of IGP and altered duodenal barrier function induced by acid, together with the ultrastructural studies assessing degranulation could suggest that acid do not directly activate mast cells as probably occurs in the esophagus^32^. Nevertheless, the increased amount of tryptase in the mucosa after acid perfusion suggests the opposite. Our and other studies show that the assessment of the activation of mast cells in physiological and pathophysiological conditions is complex. Secretion of mediators can occur without evidence of degranulation, and even mediators stored within the same granule can be selectively released in a discriminatory pattern^44^. Interestingly, Gottwald et al. found that electrical vagal stimulation increases histamine levels in intestinal tissues without degranulation of mast cells^45^. Furthermore, IL-1 stimulates secretion of IL-6 from mast cells without release of tryptase^46^. These and other data suggest the possibility of activation/modulation of mast cells without degranulation. Whether the specific synthesis and release of certain mediators without decrease in granular content can be blocked by DSCG is unknown. Further experiments are needed to confirm that duodenal acid perfusion activates mast cells in healthy subjects. Nevertheless, irrespective of the outcome of these studies, mast cell activation does not seem to be involved in the acid-induced barrier defect based on our human and mice studies.

Limitations of the study include the fact that the study was performed in HV and not in patients and that, the acid perfusion was set at a short perfusion time to limit the amount of acid infused. Moreover, our data cannot demonstrate that DSCG treatment sufficiently stabilized the mast cells are since tryptase expression levels were similar between the active and the placebo arm. Nevertheless, we previously used the same dosing and treatment duration in another study where DSCG counteracted the effect of stress on small intestinal permeability during psychological stress^41^. However, we cannot exclude a potential effect of more potent mast cell stabilizers such as ketotifen or blockers of mast cell products such as histamine, e.g. the histamine receptor 1-blocker ebastine.

In conclusion, we demonstrated that duodenal acid perfusion in HV decreases IGP, disrupts epithelial integrity and promotes tryptase production in mucosal mast cells. An increased duodenal acid exposure could therefore underlie gastric dysfunction, altered duodenal permeability and low-grade inflammation observed in FD and can thus be considered a potential pathophysiological mechanism contributing to dyspeptic symptom generation. This study additionally suggests that mast cell activation is not implicated in activation of the duodenogastric reflex and increased permeability resulting from duodenal acidification. Our data support further evaluation of duodenal acid as a therapeutic target in FD but oppose the idea of using DSCG as a possible treatment in acid-induced duodenal barrier dysfunction and gastric dysmotility.

## Material and methods

### Study subjects

Healthy volunteers were recruited from a mailing list after exclusion of gastrointestinal symptoms or a history of gastrointestinal disease and were included in a double-blind, randomized, cross-over study. Exclusion criteria were regular use of medication besides oral contraceptives, type 1 or 2 diabetes or first-degree family members with type 1 diabetes, celiac disease or inflammatory bowel disease. Non-steroidal anti-inflammatory drugs were not allowed in the month before and alcohol in the last 3 days before the study procedures. Written informed consent was obtained prior to inclusion in the study and the human ethical committee of the University of Leuven approved the protocol. All methods were performed in accordance with our institution guidelines and regulations. The study in healthy volunteers was registered on https://www.clinicaltrials.gov as NCT02664051 (registered in 26/01/2016).

### Ex vivo study in mice

Cpa3Cre/+ gene-targeted mice have been described previously^47^. Mice were kept at the KU Leuven animal facility under SPF conditions. All experimental procedures were approved by the Animal Ethics Committee of the Medical Faculty of the KU Leuven (Leuven, Belgium). All methods were performed in accordance with our institution guidelines and regulations.

### Study design

#### Part 1: randomized cross-over acid or saline perfusion study

For the first part of this study, an assembly including a pH electrode with an antimony pH sensor and a thin infusion tube (2 mm diameter) was introduced transnasally and positioned in the second portion of the duodenum after an overnight fast. The pH electrode was calibrated using commercial buffer solutions at pH 7.0 and pH 4.0 before insertion. Duodenal pH was continuously monitored during the study period and recorded using an ambulatory data-logger (MicroDigitrapper; Synectics Medical, Stockholm, Sweden). Subsequently, a high-resolution manometry (HRM) catheter (36 channels spaced 1 cm apart; Manoscan 360, Sierra Scientific Instruments, Los Angeles, California, USA) was inserted through the nose and positioned in the gastric fundus to measure intragastric pressure (IGP) as a read-out of gastric relaxation or contraction. This method has been developed in our lab as a minimally invasive alternative to the barostat^48^. After a stabilization period of 20 min, one investigator (TV) started the infusion of 0.1 N HCl (acid) or saline in the duodenum at a rate of 5 mL min^−1^ during 30 min, in a randomized, cross-over manner. The solutions were prepared in the absence of the participant and the perfusion bags looked identical. The order of perfusion was based on an automatically generated random sequence. Perfusions were done with at least 2 weeks in between as it is known that the intestinal epithelium takes 4–7 days to renew^49^. The participants and a second investigator (HV), who was taking care of the ex vivo experiments and analyses, were blinded to the nature (acid or saline) of the infusion. During the perfusion, occurrence of symptoms (fullness, bloating, belching, nausea, satiation, epigastric burning and epigastric pain) was scored using a 100-mm visual analogue scale (VAS) before and every 5 min during the perfusion. Thirty minutes after perfusion, endoscopic duodenal biopsies were obtained (see below).

#### Part 2: randomized cross-over acid perfusion study with mast cell stabilization or placebo

For the second part of this study, a second group of participants was treated with oral placebo (190 mg mannitol) or disodiumcromoglycate (DSCG; Nalcrom, Italchimici SpA, Rome, Italy), a mast cell stabilizer, 200 mg qid for 2 weeks^41^. Between both treatments, there was a washout period of at least 2 weeks. The order of the treatment was based on an automatically generated random sequence. Capsules and packaging of placebo and DSCG looked identical. Participants and the investigator performing the ex vivo experiments and analyses were blinded to the nature of the treatment. After treatment, the study design was as described in part 1 except that acid perfusions were performed at both study visits.

### Duodenal biopsies

Biopsy specimens were taken with a standard biopsy forceps in the second part of the duodenum by an experienced endoscopist (JT) during an esophagogastroduodenoscopy. Three biopsies were put in ice-cold oxygenated Krebs–Ringer bicarbonate buffer for Ussing chamber experiments to assess epithelial barrier function. Two biopsies were placed in RNA*later* solution (Qiagen, Hilden, Germany) for RNA isolation and real-time reverse transcriptase polymerase chain reaction (RT-PCR), to assess gene expression. Two biopsies were snap frozen in liquid nitrogen for further protein extraction and identification by western blot. One biopsy was fixed in formalin and embedded in paraffin for immunofluorescence and immunohistochemistry for specific histological analysis. Another biopsy was fixed with 2.5% (v/v) glutaraldehyde (Sigma-Aldrich, St. Louis, Missouri, USA) and 2% (v/v) paraformaldehyde (Sigma-Aldrich) in phosphate buffer at pH 7.4 for ultrastructural evaluation by transmission electron microscopy (TEM).

### Experimental methods

#### Ussing chamber experiments

Duodenal biopsies were mounted in modified 3 mL Ussing chambers (Mussler Scientific Instruments, Aachen, Germany) as described previously^5^. TEER was recorded every 30 min during 2 h. Passage through the biopsy was evaluated with the paracellular probe fluorescently labelled dextran (FITC-dx4; MW = 4000 Da, 1 mg mL^−1^; Sigma-Aldrich, St. Louis, USA). FITC-dx4 was added to the mucosal compartment and serosal samples were collected every 30 min during 2 h, of which the fluorescence level was measured using a fluorescence reader (FLUOstar Omega; BMG Labtech, Ortenberg, Germany). The average values of time points 60, 90 and 120 min was taken. Results of TEER and FITC-dx4 passage were presented as values relative to the mean of the control group.

#### RNA isolation, c-DNA synthesis and quantitative real time PCR

Real-time reverse transcription polymerase chain reaction (RT-PCR) was performed as we previously described^5^ and it is described in detail in the Supplementary Methods. Primer sequences are specified in Supplementary Table S2.

#### Western blot

Western blot was performed as we previously described^5^. Equal amounts of protein per sample were separated by sodium dodecyl sulfate–polyacrylamide gel electrophoresis and transferred to a polyvinylidene difluoride membrane. Blots were incubated overnight with primary antibodies: rabbit anti-CLDN1 (1:500; Abcam, Cambridge, UK), rabbit anti-CLDN2 (1:500; Abcam), rabbit anti-CLDN3 (1:500; Abcam), mouse anti-CLDN4 (1:1000; Invitrogen, Carlsbad, USA), rabbit anti-OCLN (1:1000; Invitrogen), rabbit anti-DSC2 (1:500; Abcam), mouse anti-DSG2 (1:1000; Abcam), mouse anti-tryptase (1:500; Dako, Glostrup, Denmark) or mouse anti-MBP (1:500; AbD Serotec, Kidlington, UK). All membranes were stained with mouse anti-vinculin (1:5000; Sigma-Aldrich), as a protein loading control. Peroxidase-conjugated goat anti-rabbit IgG or goat anti-mouse IgG (both 1:5000; Thermo Scientific) were used as secondary antibodies. Bands were quantified by densitometry using ImageJ software (National Institutes of Health; https://rsb.info.nih.gov.ij/). Fold change was determined relative to the average of the group perfused with the saline solution (part 1) or the placebo oral treatment (part 2).

#### Immunofluorescence

Immunofluorescence was performed as described before^5^. Deparaffinization and rehydration were performed following standard procedures using xylene and graded solutions on 5 µm sections. Tissues were then blocked with Protein Blocking Solution (Dako) and incubated during 60 min at room temperature in mouse anti-ZO-1 (1:50; Invitrogen), rabbit anti-β-catenin (1:250; Abcam) or mouse anti-E-cadherin (1:50; Abcam). Alexa Fluor 488 goat anti-mouse IgG and Alexa Fluor 594 donkey anti-rabbit IgG (both 1:1000; Invitrogen) were used as secondary antibodies. Ten representative non-overlapping confocal images were obtained with a LSM510 Meta Laser Scanning microscope at 630× magnification (Zeiss, Oberkochen, Germany). ImageJ software was used to quantify protein in a blinded manner, measuring the average and the area of fluorescence intensity at the apical pole.

#### Immunohistochemistry

Immunohistochemistry was performed as we previously described^4,5^. After deparaffinization, sections were blocked with REAL Peroxidase Blocking (Dako) and Protein Blocking Solution (Dako). Eosinophils and mast cells were stained by incubating sections at room temperature for 60 min in mouse anti-MBP (1:20) or 30 min in mouse anti-mast cell tryptase (1:200) or anti-cKit (1:250), respectively. Sections were incubated with secondary horse anti-mouse biotinylated antibody (1:200; Vector Laboratories, Burlingame, California, USA) and diaminobenzidine was used as the chromogen, followed by counterstaining with Harris’s haematoxylin. Pictures of at least seven representative non-overlapping high-power fields (HPFs) at 400× magnification were taken on an optical microscope (BX41 Olympus; Olympus, Aartselaar, Belgium) in a blinded manner. The area of the lamina propria was measured using ImageJ software and positive cells were counted. Results are expressed as positive cells per mm^2^.

#### Transmission electron microscopy

Transmission electron microscopy technique is described in detail in the Supplementary Methods.

#### Ex vivo acid exposure in mice

Duodenal tissue from mice deficient in mast cells (Cpa3^Cre/+^) and their wild type littermates was mounted in Ussing chambers as described above for duodenal biopsies. After a stabilization period of 30 min, one tissue of each mouse was exposed to HCl (pH 1.3) during 30 min, while one tissue was used as a control (krebs buffer). TEER was recorded just before acid exposure and 30 min after acid exposure.

### Data analysis

Data analysis was performed as we previously described^11,48^. The severity VAS scores of each symptom during perfusion were averaged and corrected for the score before the perfusion. For IGP measurements, an interpolated thermal compensation was done on the recording to correct for thermal drift during the measurement. The original data were exported from the recording software (Manoview Analysis, Sierra Scientific Instrument, Los Angeles, USA) to Microsoft Excel. To avoid influences on IGP from movement, coughing, swallowing or sneezing, a moving median was calculated per channel over a 30 s frame. Per channel, a baseline value was calculated from the moving median data as the average pressure in the last 5 min of the stabilization period. Data were presented per minute as the difference of the minimum moving median value in that minute and the baseline value of the five selected channels below the lower esophageal sphincter. The area under the curve (AUC) at each minute was calculated and averaged over the 30 min perfusion period.

### Statistical analysis

Differences between groups were analyzed using paired Student’s t-tests or Wilcoxon signed rank tests when appropriate and data are presented as mean ± SEM or median (IQR) respectively. Differences between more than two groups were analyzed using Kruskal–Wallis, followed by post-hoc testing (Dunns correction for multiple testing). All results were analyzed using SAS 9.2 (SAS Institute, Cary, USA) and values were considered statistically significant when *P* < 0.05. Bonferroni correction for multiple testing was performed.

## Supplementary information

Supplementary Information 1.

Supplementary Information 2.
