# Supplementary material for: Duodenal acidification induces gastric relaxation and alters epithelial barrier function by a mast cell independent mechanism
Source: Sci Rep. 2020 Oct 15;10:17448. doi: 10.1038/s41598-020-74491-1 (PMC7562901; doi:10.1038/s41598-020-74491-1)
Supplement: Supplementary file 1 — Supplementary Information 1. [file 41598_2020_74491_MOESM1_ESM.docx]

**DUODENAL ACIDIFICATION INDUCES GASTRIC RELAXATION AND ALTERS EPITHELIAL BARRIER FUNCTION BY A MAST CELL INDEPENDENT MECHANISM**

**Short title:** Duodenal acidification and hyperpermeability

Hanne Vanheel^1^, Maria Vicario^2,4,#^, Dorien Beeckmans^1^, Silvia Cocca^3^, Lucas Wauters^1,5^, Alison Accarie^1^, Joran Toth^1^, Hans-Reimer Rodewald^6^, Gert De Hertogh^7^, Gianluca Matteoli^1^, Guy Boeckxstaens^1^, Jan Tack^1,5^, Ricard Farré ^1,4*^, Tim Vanuytsel^1,5^

^1^Translational Research Center for Gastrointestinal Disorders, Department of Chronic Diseases, Metabolism and Ageing, KU Leuven, Leuven, Belgium; ^2^Digestive Diseases Research Unit, Department of Gastroenterology, Institut de Recerca Vall d’Hebron, Hospital Universitari Vall d’Hebron, Universitat Autònoma de Barcelona, Barcelona, Spain; ^3^Department of Digestive Diseases, Campus Bio-Medico University, Rome, Italy. Endoscopy Unit-Azienda Ospedaliero Universitaria di Modena, Italy; ^4^Centro de Investigación Biomédica en Red de Enfermedades Hepáticas y Digestivas (CIBERehd), Instituto de Salud Carlos II, Madrid, Spain. ^5^Department of Gastroenterology and Hepatology, University Hospitals Leuven, Leuven, Belgium; ^6^Division of Cellular Immunology, German Cancer Research Center, Heidelberg, Germany; ^7^Department of pathology, University Hospitals Leuven, Leuven, Belgium.

^#^Current address: Department of Gastrointestinal Health, Société des produits Nestlé S.A., Nestlé Research, Vers-chez-les-Blanc, 1000 Lausanne 26, Switzerland

^*^ Shared senior authorship

**Supplementary Methods**

**RNA isolation, c-DNA synthesis and quantitative real time PCR**

RNA was isolated with the RNeasy Mini Kit (Qiagen) and subsequent on-column DNase treatment using the RNase-free DNase set (Qiagen) according to the manufacturer’s specifications. cDNA synthesis was performed using 2µg of RNA mixed with qScript cDNA SuperMix (Quanta Biosciences, Gaithersburg, USA). Quantitative RT-PCR was executed using LightCycler 480 SYBR Green I Master Mix (Roche Applied Science, Penzberg, Germany) and primers for claudin (CLDN) 1-4, occludin (OCLN), zonula occludens (ZO) 1-3, β-catenin, E-cadherin, desmocollin-2 (DSC2), desmoglein-2 (DSG2), tryptase (TPSAB1) and eosinophil major basic protein (PRG2) (all from TIB Molbiol, Berlin, Germany; Suppl Table 1) on a LightCycler 480 (Roche Applied Science). To normalize the mRNA expression, the human 18S ribosomal RNA gene (S18) was used as a housekeeping gene. Data were analyzed using the 2^-ΔΔCt^ method as previously described [48]. Fold change was calculated individually with respect to the group perfused with the saline solution (part 1) or the group receiving the placebo treatment (part 2).

**Transmission electron microscopy**

Duodenal samples were processed for ultrastructural analysis as we previously described [4]. Briefly, after fixation duodenal biopsies were post-fixed in 1% (w/v) osmium tetroxide containing 0.8% (w/v) (Sigma-Aldrich) at 4 °C. Samples were dehydrated, infiltrated in Epon’s resin and polymerized. Ultrathin sections (70 nm) were mounted in copper grids, contrasted and observed in a Jeol JEM-1400 TEM equipped with a Gatan Ultrascan ES1000 CCD camera (Jeol LTD, Tokyo, Japan). Examinations were performed independently by one experienced investigator (MV) in a blinded manner on a minimum of 30 sections per biopsy sample. The general structure of each tissue was evaluated, and only samples containing both intact epithelium and lamina propria (assuring good fixation) were evaluated. Cell morphology, integrity of the epithelium and the intercellular space were evaluated based on visual analysis. Mast cells were identified based on their specific cytoplasmic granules morphology. The analysis was performed as previously reported [4]. Densitometric analysis of the cytoplasmic granules of mast cells, as indicative of the content release (degranulation), was performed using ImageJ. Regions of interest were drawn over individual granules to determine their pixel intensity profile and average intensity (arbitrary units). All analyses were performed in a blinded manner.

**SUPPLEMENTARY TABLES**

**Supplementary table S1:** Dyspeptic symptoms, evaluated by a 100mm visual analogue scale, during saline and acid perfusion.

| **Symptom** | **Saline** | **Acid** | ***P* value** |
| --- | --- | --- | --- |
| Fullness | 0 (0-0) | 0 (0-0) | 0.63 |
| Bloating | 0 (0-0) | 0 (-2.25-0.88) | 0.81 |
| Belching | 0 (0-0) | 0 (0-0.88) | 0.81 |
| Nausea | 0 (0-0) | 0 (0-2.75) | 0.13 |
| Satiation | 0 (0-0) | 0 (0-0.13) | 1.00 |
| Epigastric Burning | 0 (0-0) | 0 (0-0.38) | 0.38 |
| Epigastric Pain | 0 (0-0) | 0 (0-0.25) | 0.38 |

Data are median (IQR)

**Supplementary table S2:** Primer sequences

Forward Reverse

CLDN1 GGGCTGCAGCTGTTGGGCTT GGGTTGCTTGCAATGTGCTGCT

CLDN2 GGCGGTAGCAGGTGGAGTC CTTGGTAGGCATCGTAGTAGTTGG

CLDN3 CGCGGCGAGAGCGTATGGA GGCAACGCGCAGCACACGAT

CLDN4 CCCCGAGAGAGAGTGCCCTG AGCGTCCACGGGAGTTGAGGA

OCLN TGTGGATCCCCAGGAGGCCA AGGCACGTCCTGTGTGCCTG

ZO1 CAAGATAGTTTGGCAGCAAGAGATG ATCAGGGACATTCAATAGCGTAGC

ZO2 GCCCCAGGCATGGAAGAGCTG CCCACCCGGGAGCACATCAGA

ZO3 CAGCCAGACCGACTCTCCCGT GGGGCTGTACCCACGATCCTC

β-catenin AGGTGTGGCGACATATGCAGCT GGGGTTCTCCCTGGGCACCA

E-cadherin CACCTGGAGAGAGGCCGCGT AACGGAGGCCTGATGGGGCG

DSC2 TGGGCATAGCATTGCTCTTTTGCAT CCCTGAGCAGAAGCGCCCAC

DSG2 CCCGCAGCAATTGCGCTCAT TGGCAGAAATGATGGCACCACCTTG

PRG2 CTCTGGGTGGGATAAAGCCAA AGCGTCTTAGCACCCAAAGG

TPSAB1 TGTCCCCAAAAAGCCGTGAG TTTGGACAGCAGGGGTTGGT

S18 ACCAACATCGATGGGCGGCG TGGTGATCACACGTTCCACCTCA

CLDN, claudin; OCLN, occludin; ZO, zonula occludens; DSC2, desmocollin-2; DSG2, desmoglein-2; PRG2, eosinophil major basic protein; TPSAB1, tryptase Alpha/Beta 1; S18, ribosomal protein S18.
