# Supplementary material for: Duodenal acidification induces gastric relaxation and alters epithelial barrier function by a mast cell independent mechanism
Source: Sci Rep. 2020 Oct 15;10:17448. doi: 10.1038/s41598-020-74491-1 (PMC7562901; doi:10.1038/s41598-020-74491-1)

DUODENAL ACIDIFICATION INDUCES GASTRIC RELAXATION AND ALTERS EPITHELIAL BARRIER FUNCTION BY A MAST CELL INDEPENDENT MECHANISM

Short title: Duodenal acidification and hyperpermeability

Hanne Vanheel[1], Maria Vicario[2,4,#], Dorien Beeckmans[1], Silvia Cocca[3], Lucas Wauters[1,5], Alison Accarie[1], Joran Toth[1], Hans-Reimer Rodewald[6], Gert De Hertogh[7], Gianluca Matteoli[1], Guy Boeckxstaens[1], Jan Tack[1,5], Ricard Farré [1,4*], Tim Vanuytsel[1,5]

[1]Translational Research Center for Gastrointestinal Disorders, Department of Chronic Diseases, Metabolism and Ageing, KU Leuven, Leuven, Belgium; [2]Digestive Diseases Research Unit, Department of Gastroenterology, Institut de Recerca Vall d'Hebron, Hospital Universitari Vall d'Hebron, Universitat Autònoma de Barcelona, Barcelona, Spain; [3]Department of Digestive Diseases, Campus Bio-Medico University, Rome, Italy. Endoscopy Unit-Azienda Ospedaliero Universitaria di Modena, Italy; [4]Centro de Investigación Biomédica en Red de Enfermedades Hepáticas y Digestivas (CIBERehd), Instituto de Salud Carlos II, Madrid, Spain. [5]Department of Gastroenterology and Hepatology, University Hospitals Leuven, Leuven, Belgium; [6]Division of Cellular Immunology, German Cancer Research Center, Heidelberg, Germany; [7]Department of pathology, University Hospitals Leuven, Leuven, Belgium.

[#]Current address: Department of Gastrointestinal Health, Société des produits Nestlé S.A., Nestlé Research, Vers-chez-les-Blanc, 1000 Lausanne 26, Switzerland

[*] Shared senior authorship

Entire gel of the western blot for CLDN1 (figure 3B)

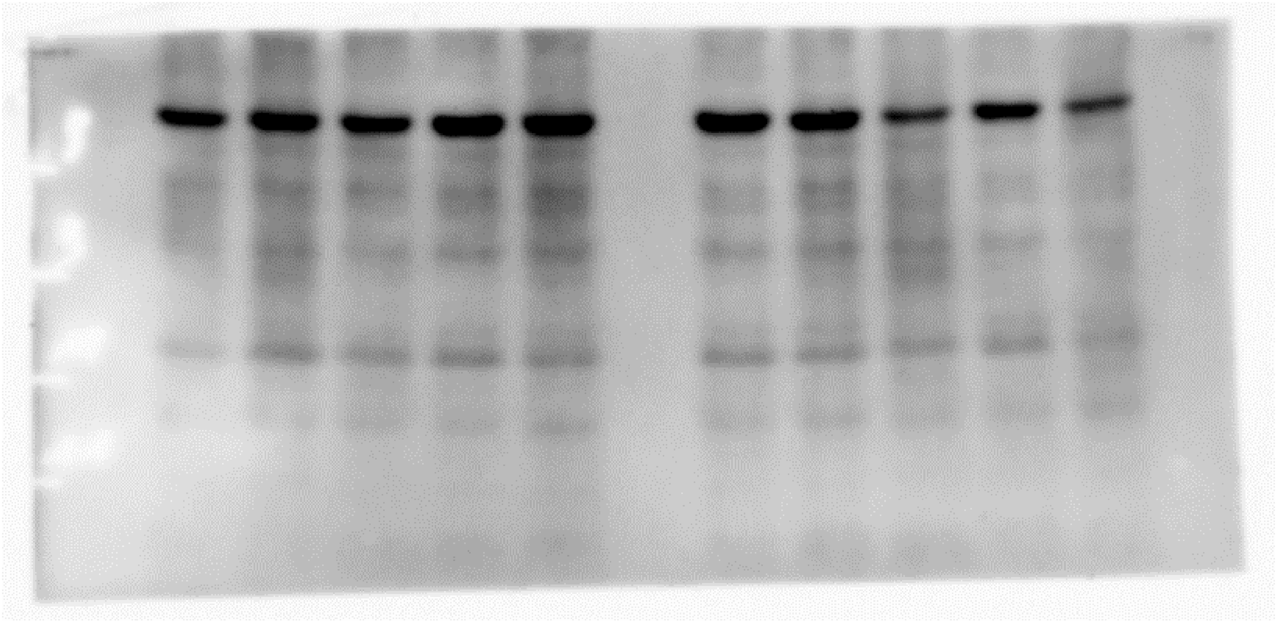

Entire gel of the western blot for CLDN2 (figure 3B)

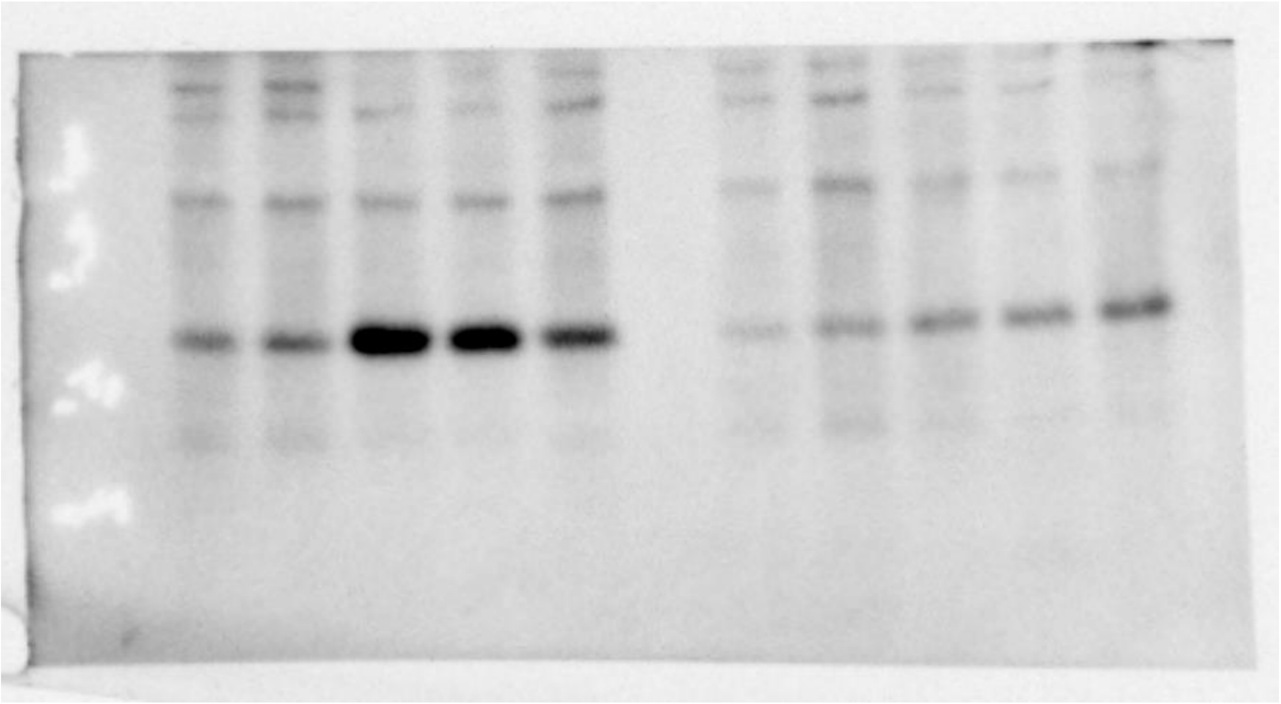

Entire gel of the western blot for CLDN3 (figure 3B)

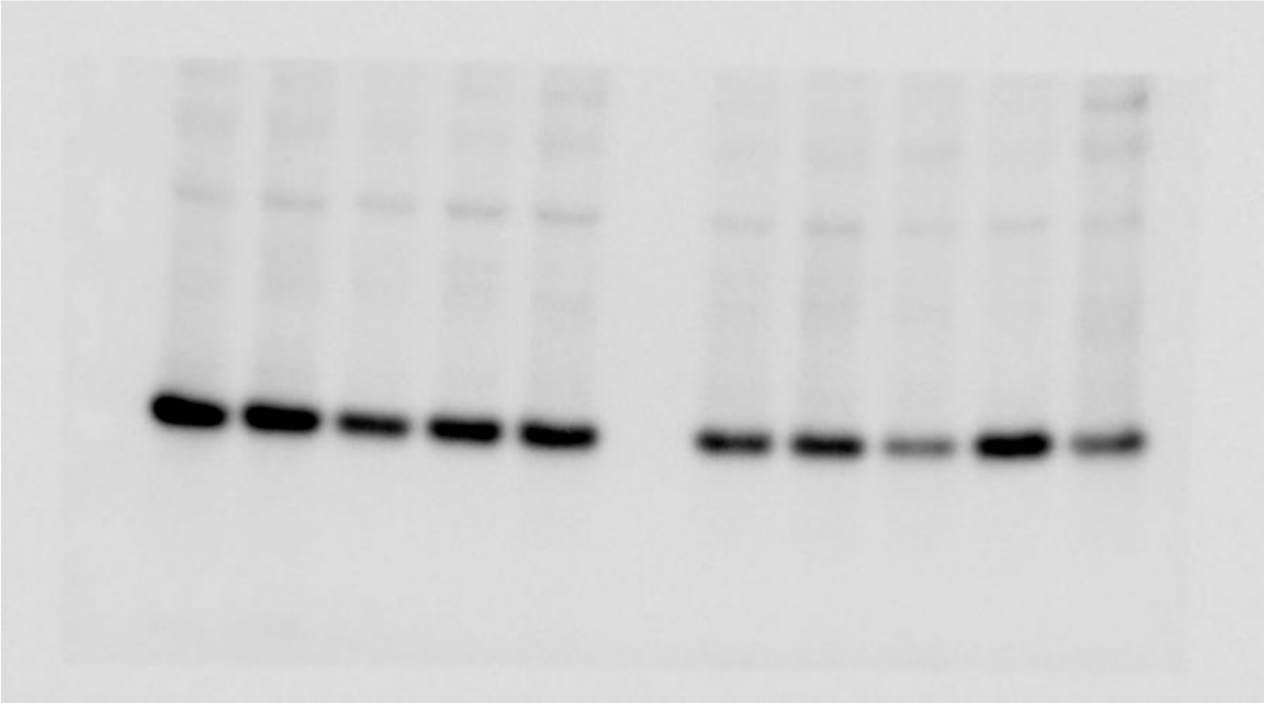

Entire gel of the western blot for CLDN4 (figure 3B)

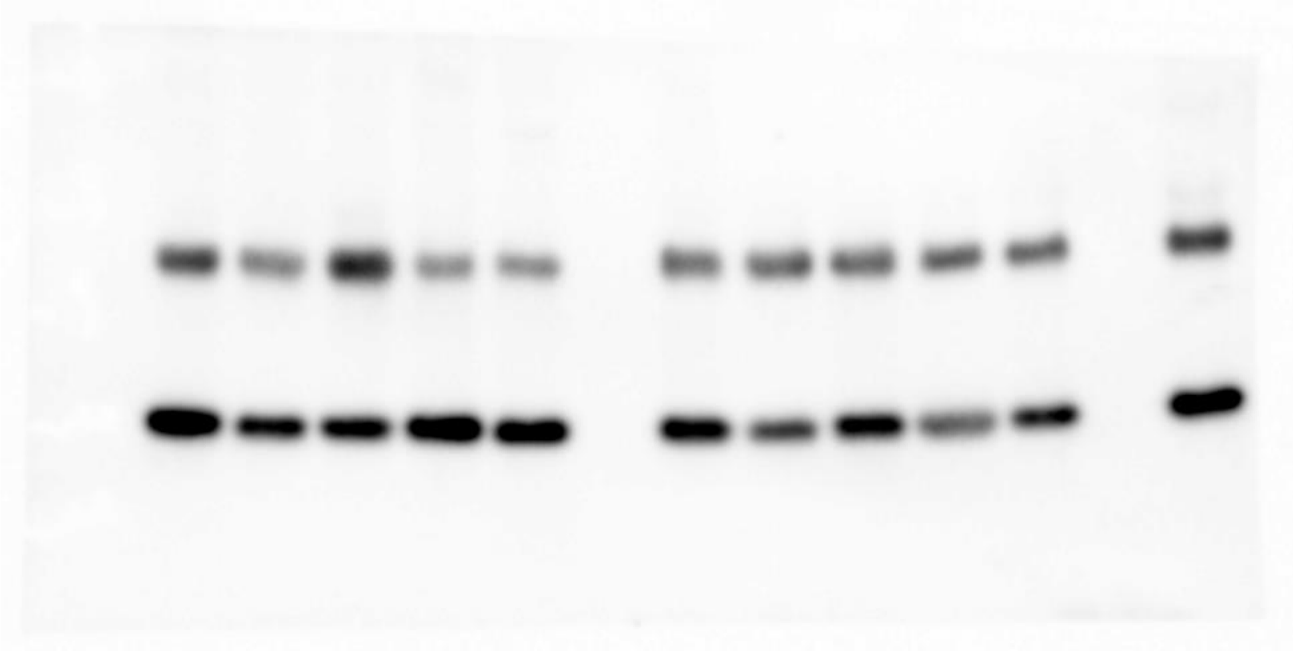

Entire gel of the western blot for OCLN (figure 3B)

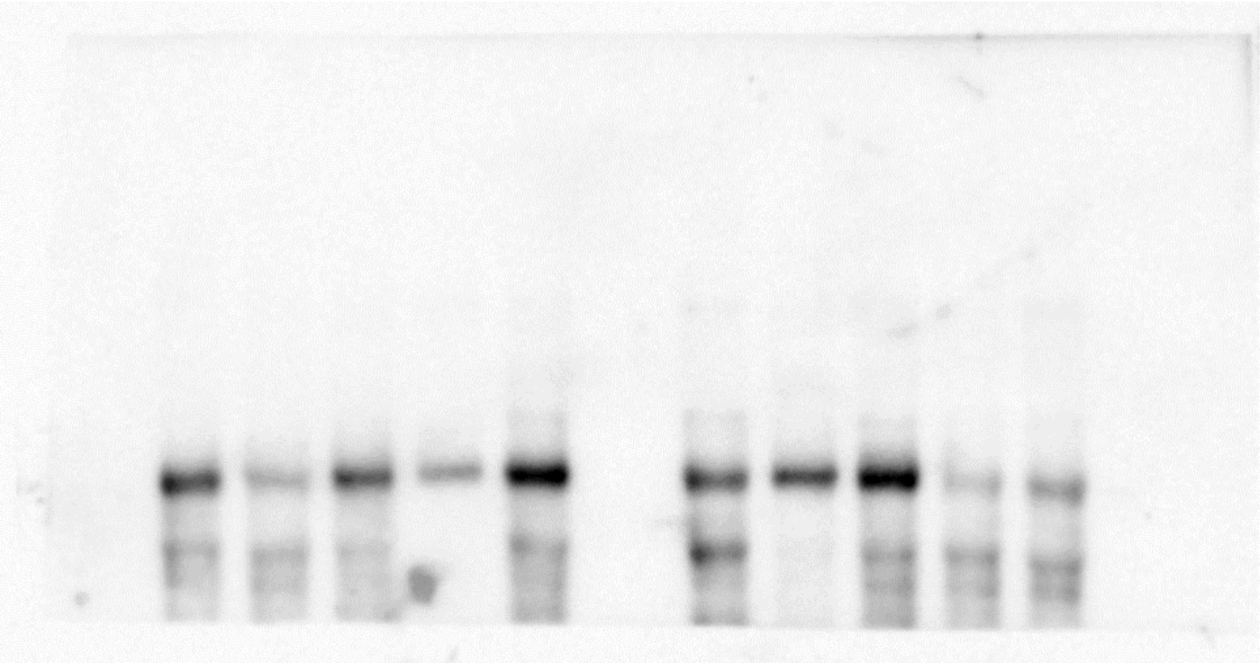

Entire gel of the western blot for DSC2 (figure 3B)

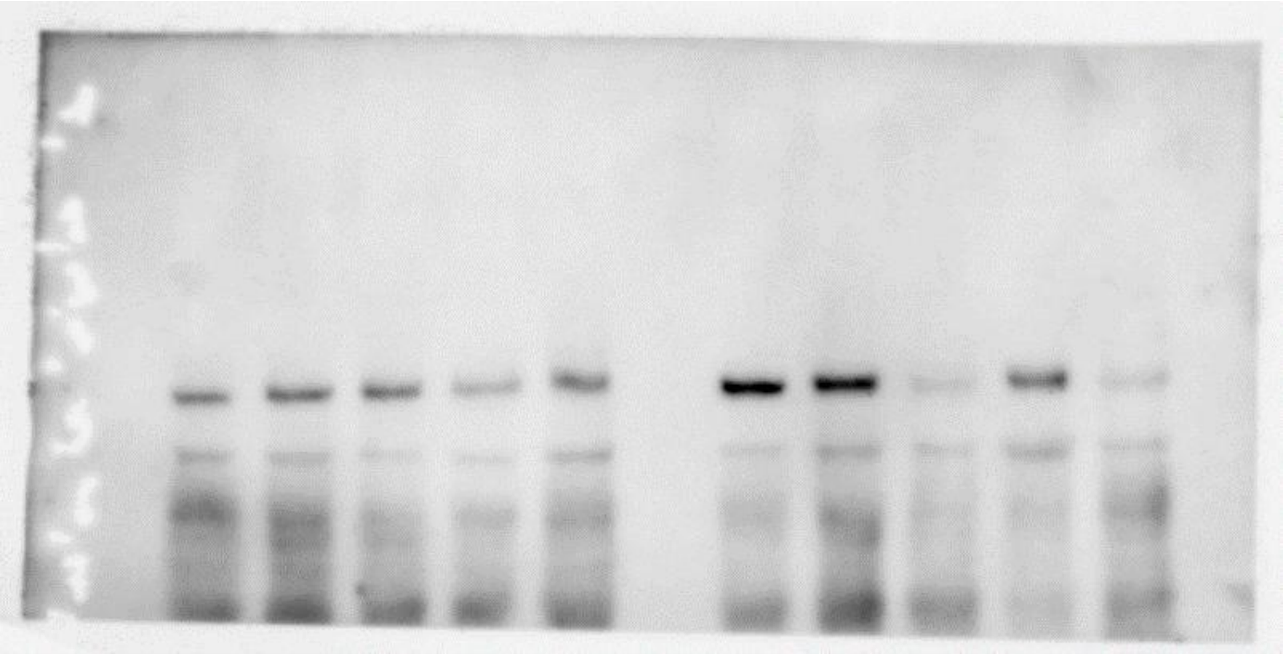

Entire gel of the western blot for DSG2 (figure 3B)

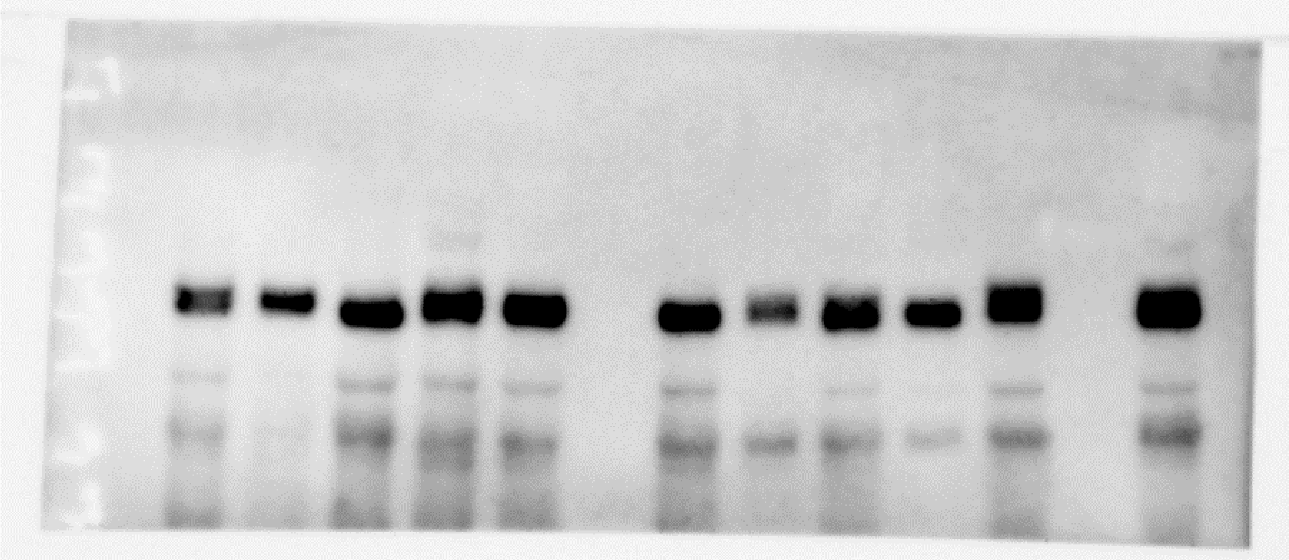

Entire gel of the western blot for vinculin (figure 3B)

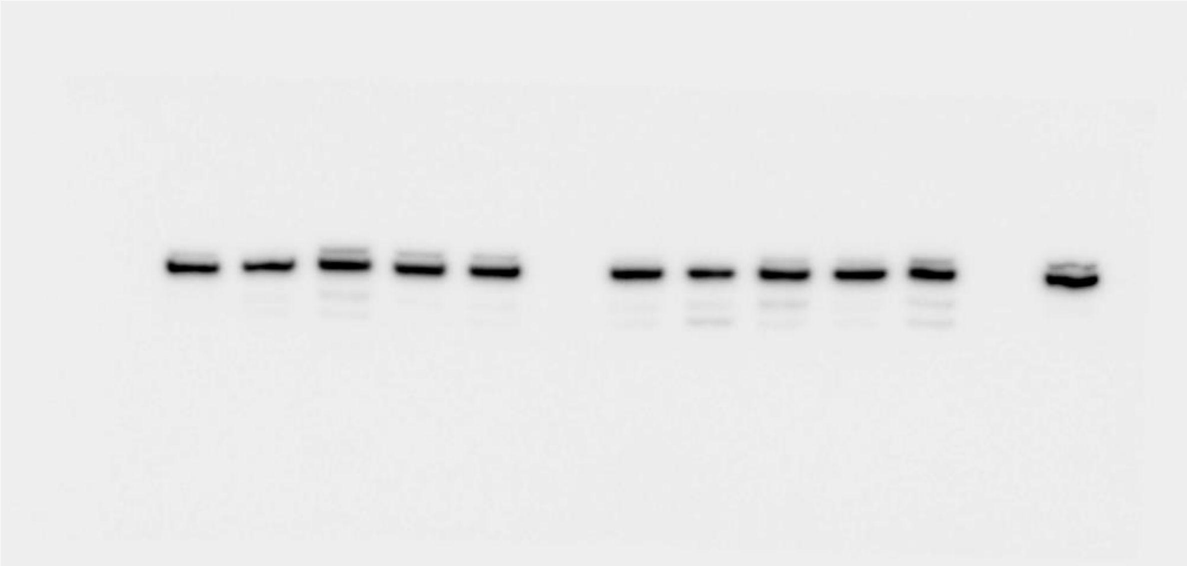

Entire gel of the western blot for MBP (figure 4F)

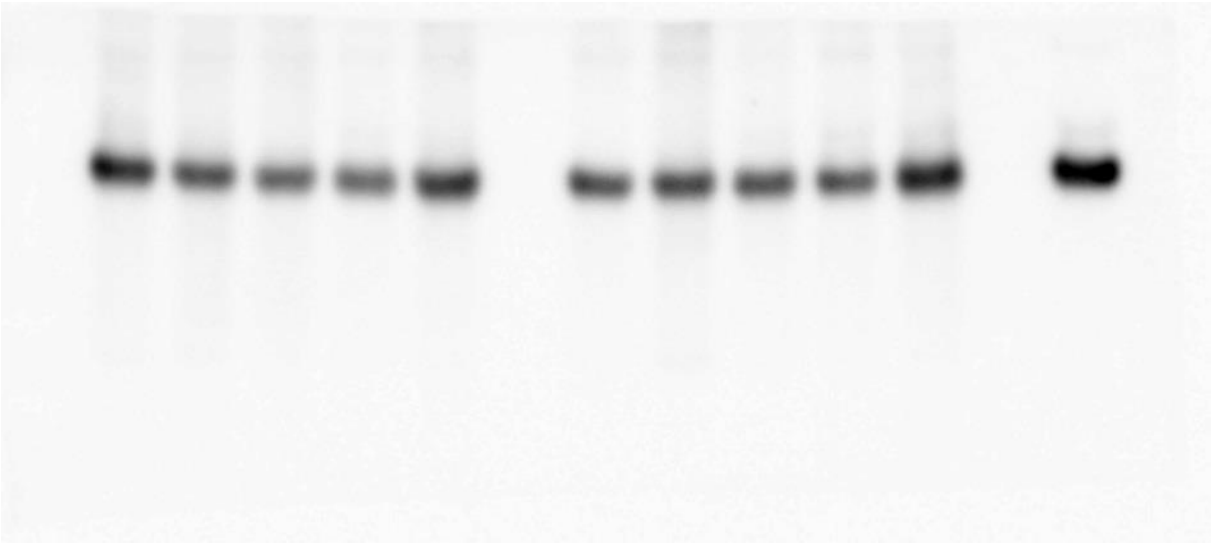

Entire gel of the western blot for tryptase (figure 4F)

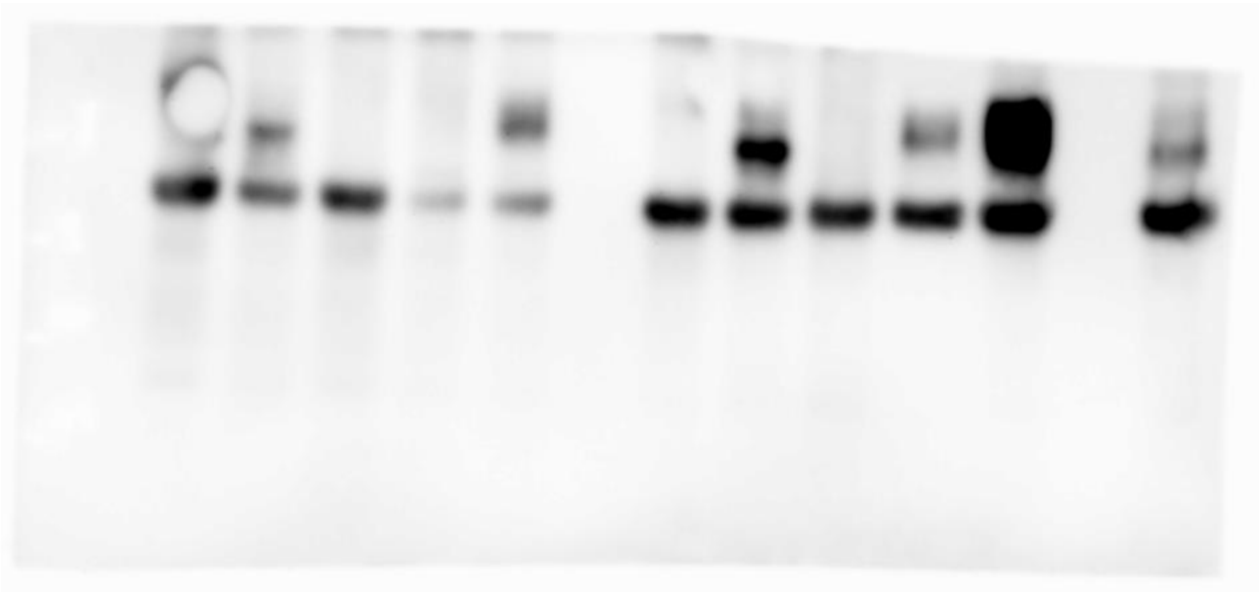

Entire gel of the western blot for vinculin (figure 4F)

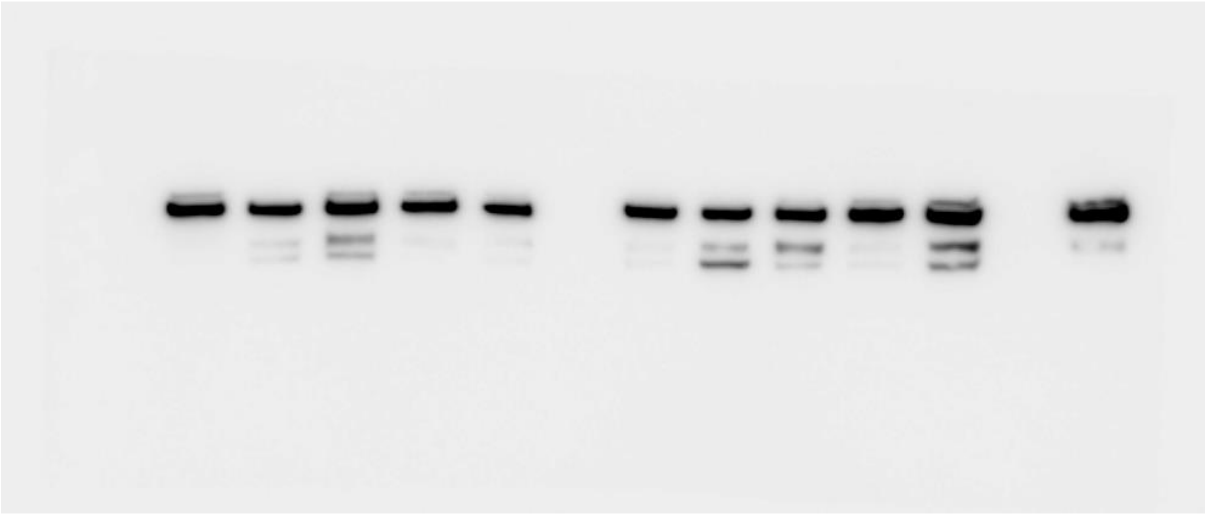

Entire gel of the western blot for MBP (figure 7F)

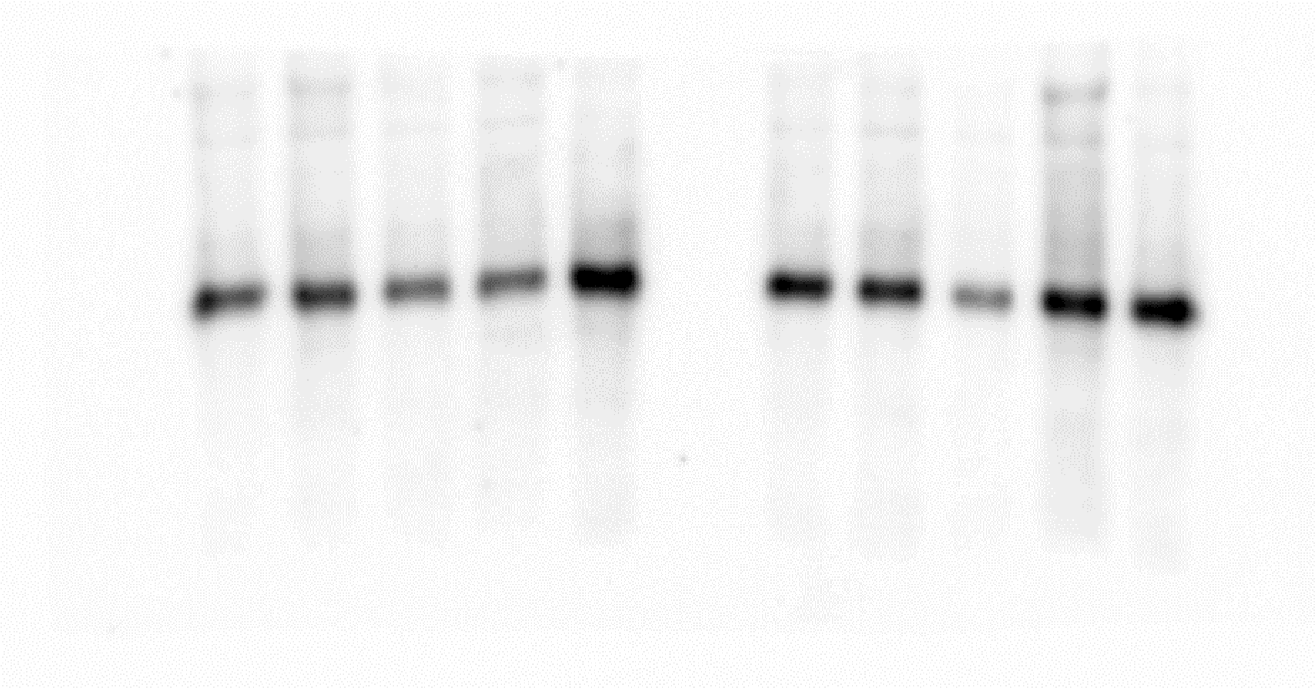

Entire gel of the western blot for tryptase (figure 7F)

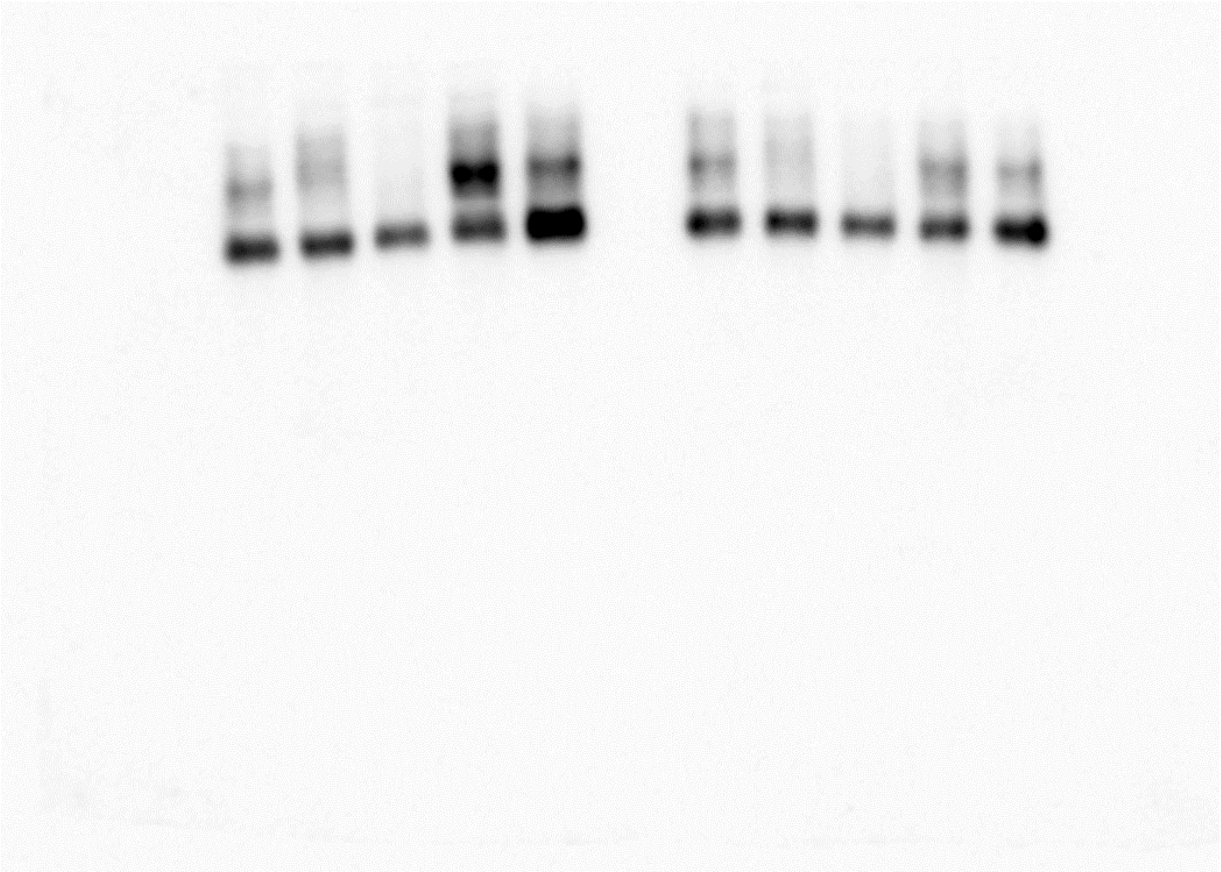

Entire gel of the western blot for vinculin (figure 7F)

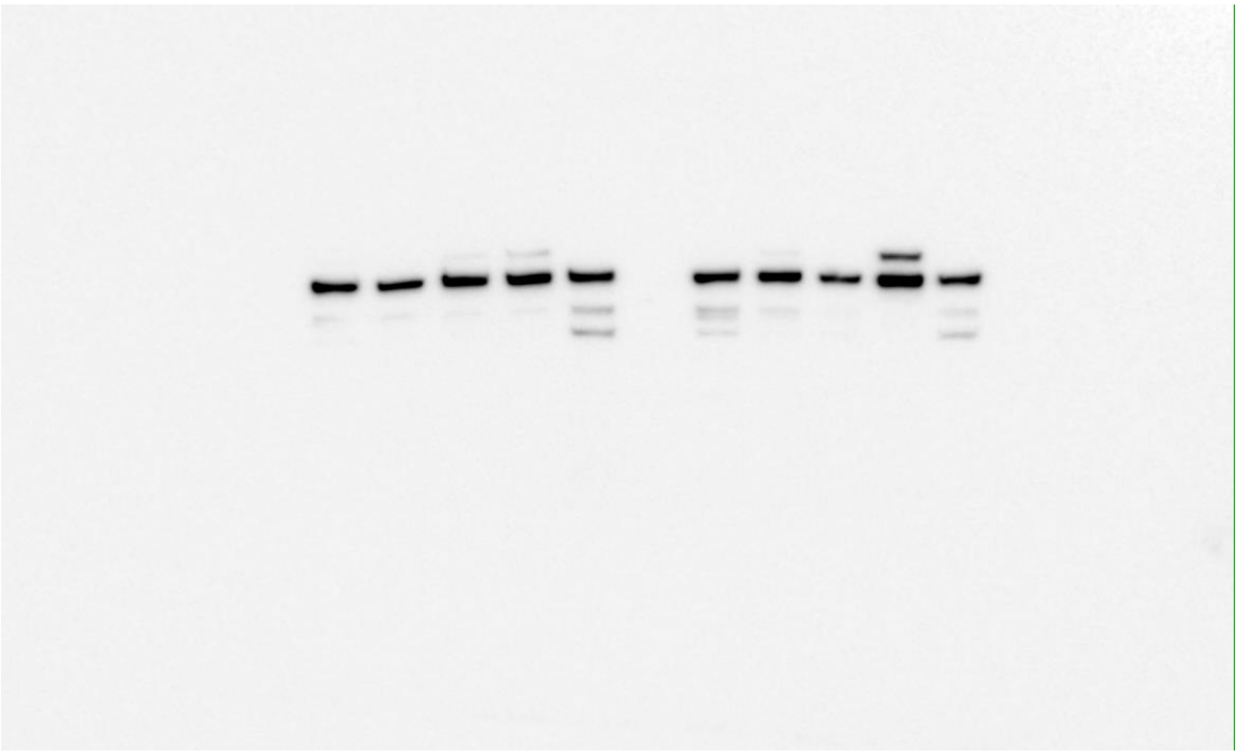

Supplement: Supplementary file 2 — Supplementary Information 2. [file 41598_2020_74491_MOESM2_ESM.pdf]
